# Altered expression profiles of microRNA families during de-etiolation of maize and rice leaves

**DOI:** 10.1186/s13104-016-2367-x

**Published:** 2017-02-24

**Authors:** Jiajia Xu, Yuanyuan Li, Yaling Wang, Xinyu Liu, Xin-Guang Zhu

**Affiliations:** 1 Key Laboratory of Computational Biology and Partner Institute for Computational Biology, Chinese Academy of Sciences, Shanghai, China; 2 State Key Laboratory of Hybrid Rice Research, Shanghai Institute of Biological Sciences, Chinese Academy of Sciences, Shanghai, China

## Abstract

**Background:**

MicroRNAs (miRNAs) are highly conserved small non-coding RNAs that play important regulatory roles in plants. Although many miRNA families are sequentially and functionally conserved across plant kingdoms (Dezulian et al. in Genome Biol 13, 2005), they still differ in many aspects such as family size, average length, genomic loci etc. (Unver et al. in Int J Plant Genomics, 2009).

**Results:**

In this study, we investigated changes of miRNA expression profiles during greening process of etiolated seedlings of *Oryza sativa* (C_3_) and *Zea mays* (C_4_) to explore conserved and species-specific characteristics of miRNAs between these two species. Futhermore, we predicted 47 and 42 candidate novel miRNAs using parameterized monocot specific miRDeep2 pipeline in maize and rice respectively. Potential targets of miRNAs comprising both mRNA and long non-coding RNA (lncRNA) were examined to clarify potential regulation of photosynthesis. Based on our result, two putative positive Kranz regulators reported by Wang et al. (2010) were predicted as potential targets of miR156. A few photosynthesis related genes such as sulfate adenylytransferase (*APS3*), chlorophyll a/b binding family protein etc. were suggested to be regulated by miRNAs. However, no C_4_ shuttle genes were predicted to be direct targets of either known or candidate novel miRNAs.

**Conclusions:**

This study provided the comprehensive list of miRNA that showed altered expression during the de-etiolation process and a number of candidate miRNAs that might play regulatory roles in C_3_ and C_4_ photosynthesis.

**Electronic supplementary material:**

The online version of this article (doi:10.1186/s13104-016-2367-x) contains supplementary material, which is available to authorized users.

## Background

miRNAs are about 21-nt long non-coding RNAs that regulate gene expression by binding to complementary sequences within their target mRNAs [3]. Although miRNAs only occupy about 0.01% of total RNA mass, their average copy numbers per cell were estimated to be higher than mRNAs [4]. The majority of verified miRNA binding targets are transcription factors [5]. Many evidences suggest that miRNAs play important roles during plant growth and development, see recent reviews by Jones-Rhoades et al. [6], Eldem et al. [7], Ameres and Zamore [8]. In addition, miRNAs were also reported to be involved with many other aspects such as siRNA biogenesis [9], signal transduction [10], plant disease [11] and environmental stress responses [12, 13].

Plant miRNAs differ from animal miRNAs in many aspects. Firstly, plant miRNA genes are often located in intergenic regions while animal miRNA genes are often located in introns or coding sequences [14]. Secondly, plant miRNA genes are generally monocistronic while animal miRNA genes are more often clustered together [15]. Thirdly, animal miRNAs tend to bind to 3′UTR regions of target transcripts while plant miRNAs follow more strict reverse complementary match with targets thus possessing limited number of targets compared with animal miRNAs [15]. Fourthly, plant and animal miRNAs also have different lengths and stabilities of precursors and involve different enzymes during the process of biogenesis and regulation [2, 3]. Furthermore, monocot and dicot miRNAs also differ in statistical features such as minimal free energies and stabilities of miRNA precursors [16].

Plant miRNAs are quite conserved across species. Through large scale survey of available sequences, expressed sequenced tags, and nonredundant nucleotides, a large number of miRNA families have been identified across different plant species, e.g. 15 conseved miRNA families in 11 plant species, at least 21 miRNA families conserved across monocot and dicots [17]. Zhang et al. [18] reported that they have identified 481 miRNAs that belong to 37 miRNA families across 71 different plant species. Based on miRBase release version 21 [19], 28 conserved miRNA families were identified between maize and rice while both species possess species-specific miRNAs. Plant miRNAs were reported to be involved in the regulation of many different developmental and metabolic processes, including responses of plants to different environmental conditions, such as temperature [12], abiotic stress [20] and salt stress [21, 22]. Examination of variations of categories and abundances of miRNAs under different environmental conditions is a common strategy to gain insights regarding the role of miRNAs during plant growth and development.

Light is a major environmental signal influencing many aspects of plant growth and development. Many earlier studies demonstrated that a large number of genes including transcription factors show dramatic changes after light induction [23, 24]. Some miRNAs have been implied to be involved in this process, e.g. *LONG 14 HYPOCOTYL 5* (*HY5*), a global regulator of light responsive transcription, was reported to regulate expressions of eight miRNA genes in *Arabidopsis* [25]. One major plant biological process heavily influenced by light is photosynthesis, which was suggested to be under the regulation of miRNAs [26]. Besides this possible role of miRNAs in regulating C_3_ photosynthesis in general, it is of special interest that miRNAs might also be involved in the regulation of C_4_ photosynthesis. The cell specific expression of C_4_ related proteins or enzymes is under multiple layers of regulation, see recent reviews on this by Hibberd and Covshoff [27] and Williams et al. [28]. Apart from *cis*-regulatory motifs that have been identified to play important role in controlling C_4_ specific expression patterns [29], post-transcriptional control, post-translational control and epigenetic control might also contribute to the cell specific accumulations of C_4_ enzymes, see a recent review by Wang et al. [30]. Though miRNAs are regarded as highly possible signals controlling the expression of C_4_ related genes, so far, experimental evidences suggesting such an association have not been established.

The present study explores the dynamic changes of expression levels of both known and candidate novel miRNAs and their predicted target genes during the greening of etiolated *Zea mays* (C_4_) and *Oryza sativa* (C_3_) leaves. We examined the possibilities of miRNAs involving with regulation of C_4_ photosynthesis. To aid this analysis, we parameterized a monocot specific miRNAs prediction pipeline. Our results indicated that although the majority of miRNA families are sequentially and functionally conserved between maize and rice, their expression profiles in response to light induction during de-etiolation process are quite different. Potential mRNA targets and mimic lncRNA targets of both known and candidate novel miRNAs were predicted. By checking the overlap of predicted targets with C_4_ cycle genes, reported regulators of C_4_ trait and photosynthesis related genes, we identified potential miRNAs that might be related to C_4_ photosynthesis.

## Results

### Deep sequencing of small RNAs

**Fig. 1:**
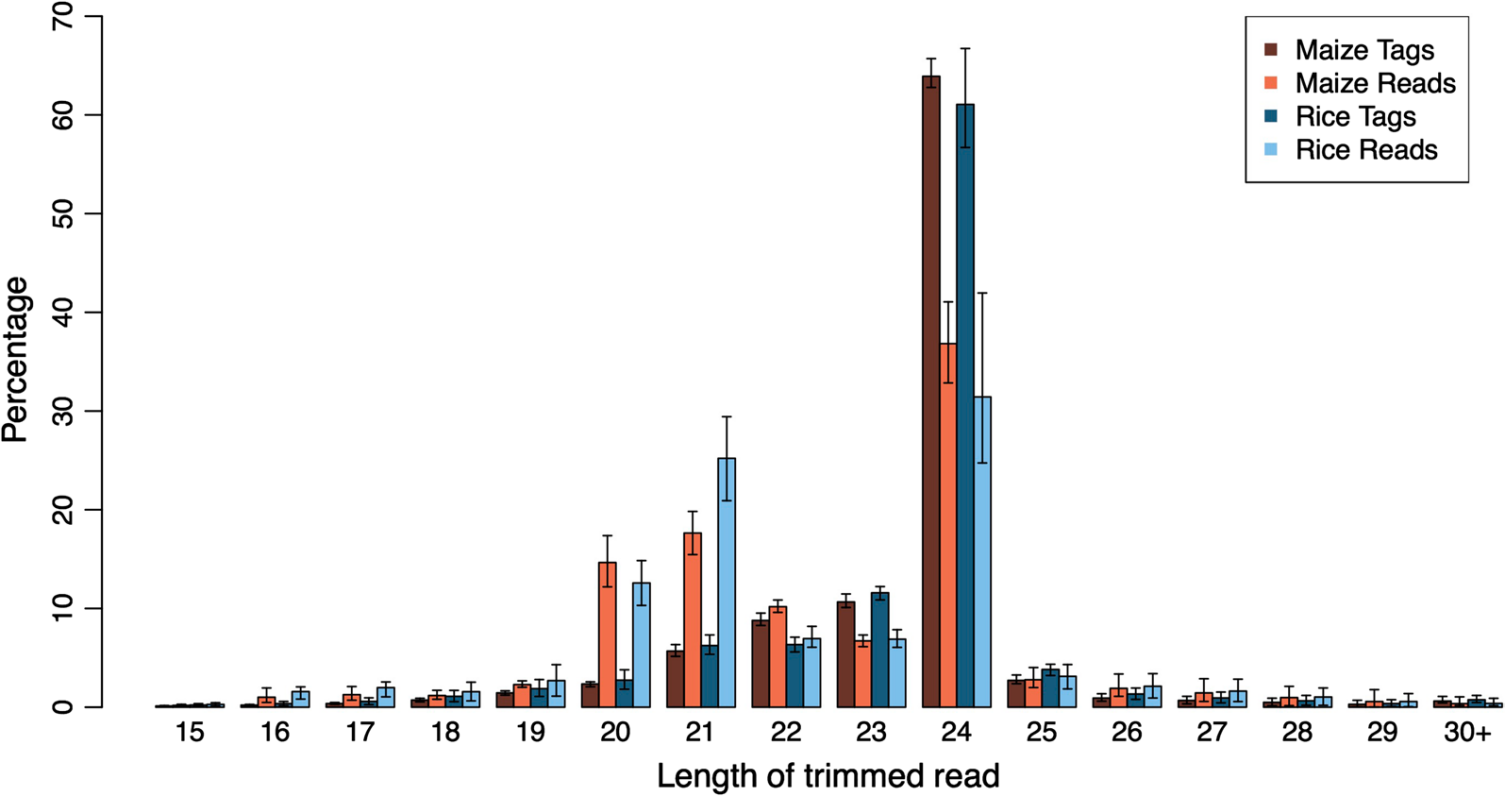
Length distribution of trimmed reads. Numbers of trimmed reads and trimmed identical reads (termed tags) were illustrated in *dark brown* (maize tags), *light brown* (maize reads), *dark blue* (rice tags) and *light blue* (rice reads). *Error bar* showed variation across 8 samples, i.e., 7 time points and 1 control sample for both maize and rice

In this study, about 85.8 and 87.5 million reads were sequenced from maize and rice small RNA libraries, which yielded about 24.2 and 19.2 million collapsed identical reads (termed tags) for maize and rice, respectively. After quality filtration, 3′ adapters were trimmed for each sample. The length distribution of the trimmed reads and tags showed that the most abundant length regions are between 20-nt and 24-nt (Fig. 1). No significant difference in length distribution between maize and rice trimmed reads was detected. Contaminant small RNA reads were removed by BLAST against rRNA, tRNA and snRNA databases (see “Methods” section for details). For each sample, numbers of reads and tags that were aligned with other small RNAs were listed in Additional file 1 and plotted in Additional file 2. In general, no significant variations between maize and rice were found in categories or amounts of identified rRNAs, tRNAs and snRNAs. Retained reads were then mapped against genomes for further analysis.

### Monocot specific miRDeep2

Plant miRNAs differ from animal miRNAs in many aspects such as biogenesis [2], precursor length [31], target binding patterns [3]. Besides, monocot and dicot plants also differ in statistical features [16]. We therefore parameterized a widely used tool miRDeep2 [32] to be monocot specific in this study. Several parameters and scoring functions were replaced in the original scripts. The modified pipeline could be used in the same way as the original one and it could be accessed via https://github.com/Rossifumi/monocot_specific_miRDeep2.git.

### Prediction of novel miRNAs in rice and maize

In total, 288 known maize miRNAs and 658 known rice miRNAs were detected in our samples. By applying the modified miRDeep2, we predicted 111 and 141 potential candidate novel miRNAs for maize and rice, respectively. Genomic loci of the precursors of these potential candidate novel miRNAs were listed in Additional file 3. Conservations of mature sequences of the predicted candidate novel miRNAs were checked across six plant species, i.e., *Arabidopsis thaliana*, *O. sativa*, *Populus trichocarpa*, *Triticum aestivum*, *Vitis vinifera*, *Z. mays*. When setting the similarity cutoff to be 90%, 6 maize and 4 rice potential miRNAs showed hits with at least one known miRNA mature sequence in miRBase (Additional file 4). Those predicted miRNAs were further filtered with expression cutoffs. A potential candidate novel miRNA was considered to be candidate novel miRNA and retained for further study only when its total TPQ (transcripts per quarter million) value in all samples together exceeded 10 (Additional file 5). Length, mature sequence, genomic loci of precursor sequence and TPQ value across samples of 47 maize candidate novel miRNAs and 42 rice candidate novel miRNAs were identified (Additional file 6).

Prior to this study, a number of genome-wide predictions of novel miRNAs in maize and rice have been conducted. For example, Wang et al. [33] predicted 167 novel miRNAs in imbibed maize seeds. Jiao et al. [34] reported 66 novel miRNAs in mixed tissues, embryo and endosperm of maize. Kang et al. [35] identified 54 novel miRNAs in developing seeds and growing leaves of maize. Sunkar et al. [36] reported 63 novel miRNAs in rice seedlings and seedlings exposed to drought and salt stresses. Here we compared our predictions with these previous studies. When setting the similarity cutoff to be 90%, 14 out of 47 maize candidate novel miRNAs were able to match with previous predictions (Additional file 7). However, no similarity was identified between 42 rice candidate novel miRNAs and the predictions from Sunkar et al. [36] at the same similarity cutoff. When lowering the similarity cutoff, we were able to identify a few matched pairs (Additional file 7). However, similarity cutoff lower than 90% is not recommanded because it would allow too many mismatches. Thus matched miRNA pairs might be quite distinguished from each other.

**Fig. 2:**
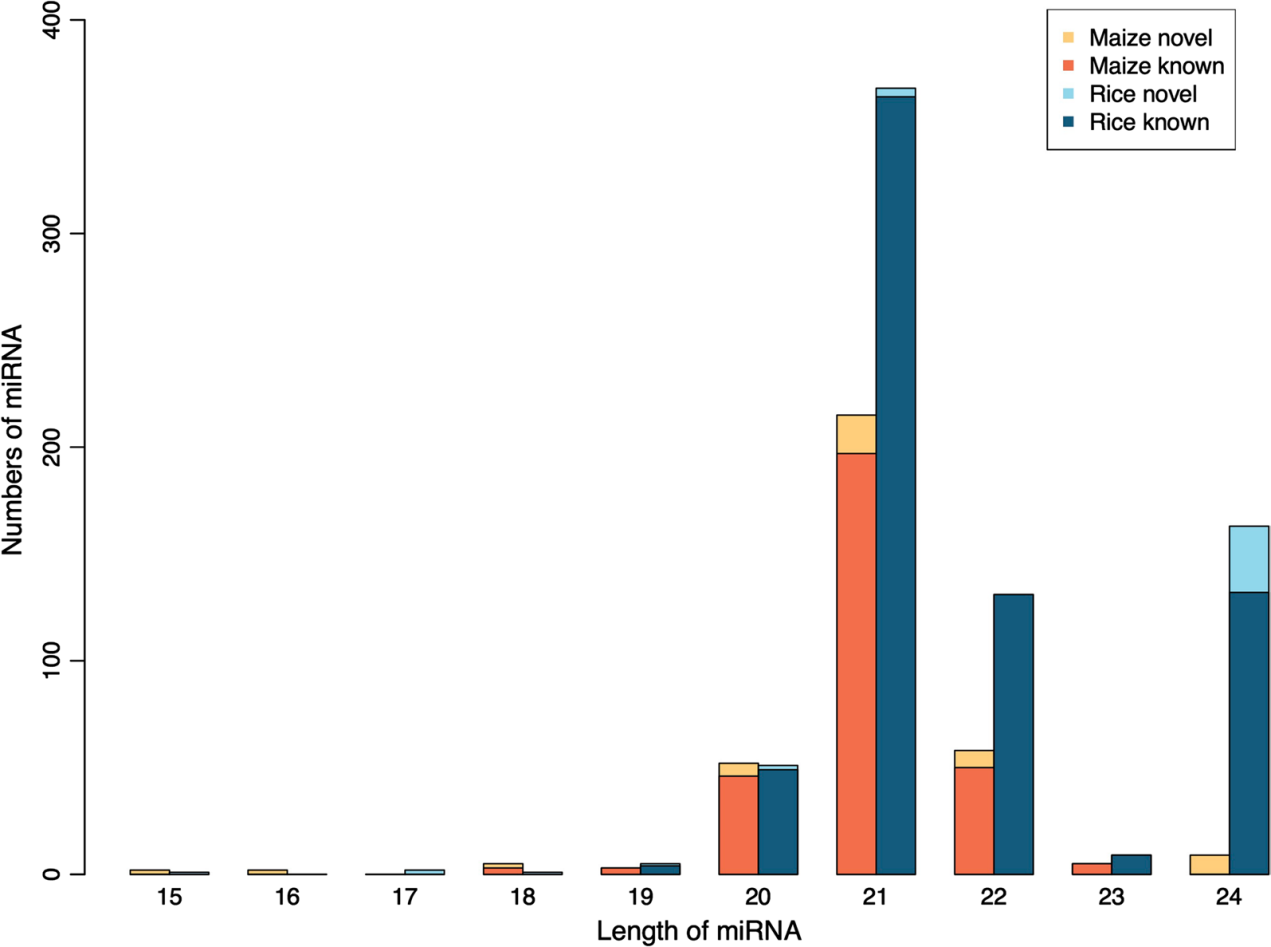
Length distribution of identified known and candidate novel miRNAs. Numbers of known and candidate novel miRNAs for maize and rice were illustrated in *dark orange*, *light orange*, *dark blue* and *light blue* respectively

The length distributions of known and candidate novel miRNAs (Fig. 2) show that although the length of the most abundant mature sequence for both maize and rice miRNAs is 21-nt, rice miRNAs however are usually slightly longer than maize miRNAs. Taking into account that the majority of plant miRNAs regulate their target mRNAs by reverse complementary match [37], the increased mature sequence length of miRNAs in rice indicate that rice miRNAs might have higher specificity in terms of target binding and thus affecting regulation.

### miRNA families and their altered expression profiles during de-etiolation

Many plant miRNA families possess multiple family members within the same species [38]. Different members from the same family are highly conserved in terms of mature sequence within or across species. However, the precursors of those miRNAs might differ at not only precursor sequence level but also their genomic loci [18]. In our study, 25 multi-member miRNA families and 3 solo-member miRNA families were detected in maize. Their homolog miRNAs were also identified in rice. Apart from these 28 conserved miRNA families between maize and rice, 30 more multi-member and 133 more solo-member miRNA families were detected in rice as well. Thus, the number of members in each miRNA family was larger in maize than rice (about 11 members per miRNA family for maize, 2 members per miRNA family for rice). Compared to rice, the majority of maize miRNAs were conserved across species while rice possess more species-specific miRNAs, according to miRBase release 21 [19].

**Fig. 3:**
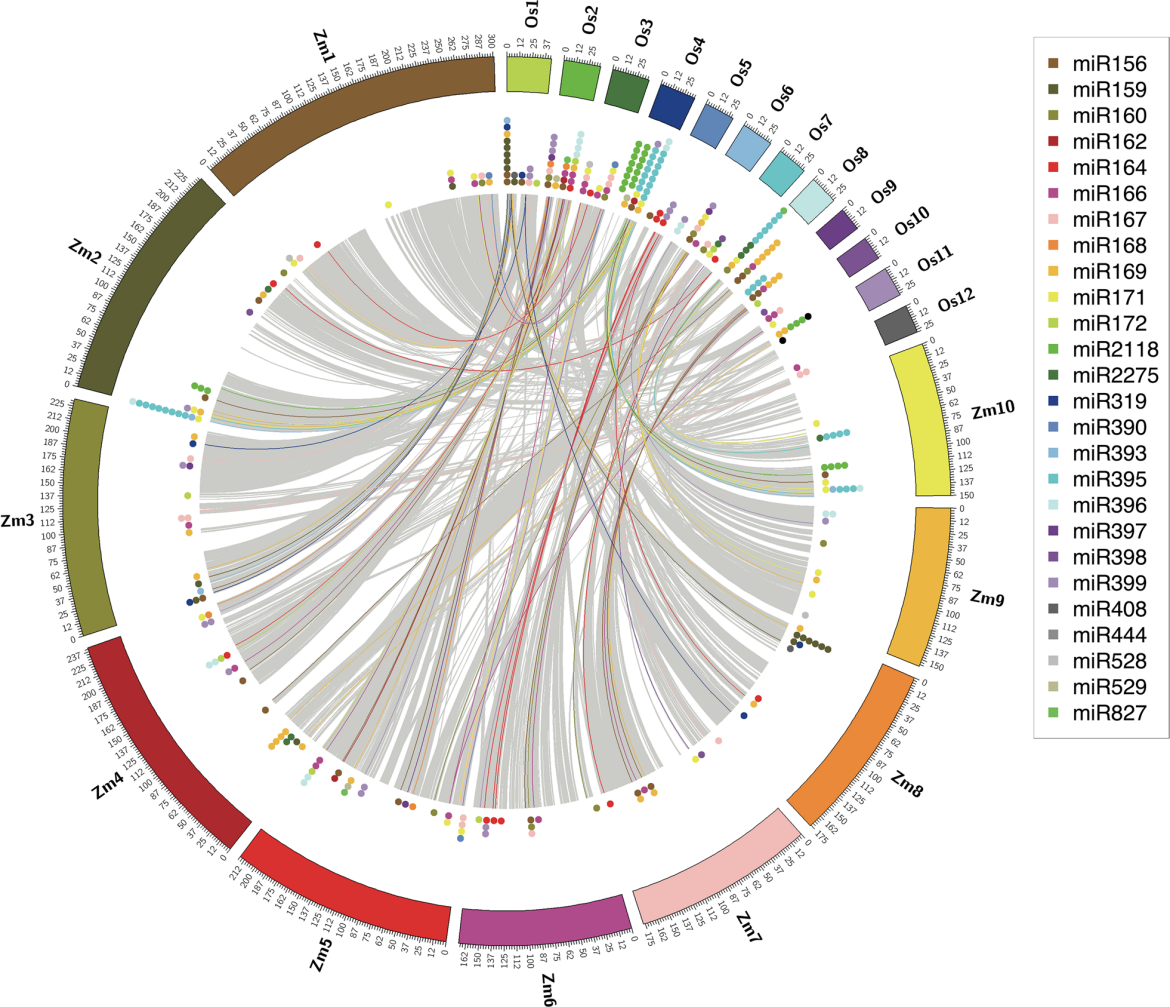
Genomic loci and syntenic gene blocks of conserved miRNAs families. *Color codes* stand for different miRNA families conserved between maize and rice. *Grey lines* linked a pair of syntenic gene blocks between these two species. *Color lines* indicated that the pair of corresponding miRNA genes resides between these two gene blocks

However, for the 28 conserved miRNA families between maize and rice, more miRNA members were detected in rice than maize (Fig. 3). Although members of plant miRNA families rarely form clusters [39], in contrast to animal miRNAs which usually cluster together [15], the conservation of genomic loci of miRNA families were also detected in plants. This is reflected in the presence of at least one member of 22 out of 28 miRNA families residing within maize and rice syntenic gene blocks (Fig. 3). More interestingly, miRNA families that form clusters in maize, such as miR2118, miR395 and miR159, tend to form clusters in rice as well, indicating that those miRNA families already existed in the last common ancestor of maize and rice.

**Fig. 4:**
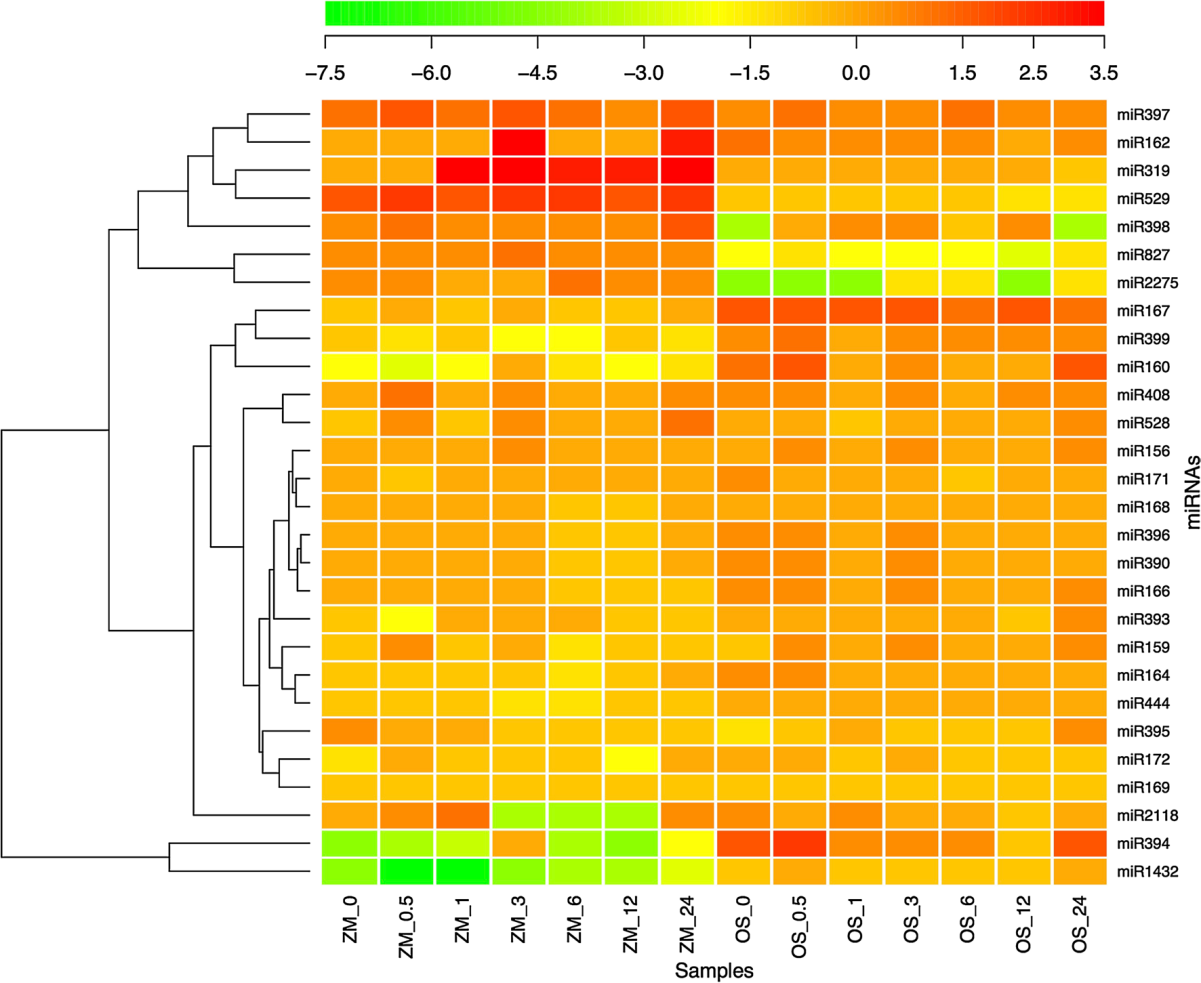
Fold-changes of expression levels of conserved miRNA families during de-etiolation. The *color bar* represents log(*e*) fold-change of TPQ (transcripts per quarter million) values of corresponding time point to control sample. If TPQ equals 0, a small value 0.001 was used instead in the calculation. Abbreviation of species name and time during de-etiolation in hours were listed in the *bottom panel*

The majority of members of the same miRNA families usually share similar mature sequences and similar regulatory roles, although a few exceptions of members of the same miRNA family with different functions were detected as well [35]. Due to sequence similarity of mature miRNAs, it is difficult to precisely detect the expression profiles of different members of the same miRNA families. To maximun the reliability of expression levels, family-wise expression profiles of 28 conserved miRNA families in maize and rice were provided in Additional file 8. Comparing the fold changes of expression values of etiolated leaves to control samples showed that although the majority of miRNA families had relatively slight changes during de-etiolation, some of them showed rather dramatic expression changes (Fig. 4).

miRNAs families with different expression patterns between maize and rice were further examined. Among miRNA families with considerable expression levels, some of them were not only differentially expressed during de-etiolation process between maize and rice but also differentially expressed between control sample and 24-h illuminated sample, i.e., their expression levels did not converge to normal condition after 24-h illumination. To be more specific, miR167 family, repressors of auxin responsive factor 8 (*ARF8*) [40], was expressed higher in rice than maize during de-etiolation process (Additional file 8). Besides, after 24-h illumination, the expression level of miR167 was still lower than control sample in maize, but significantly higher than control sample in rice (Fig. 4). Similarly, expression levels of miR159 family, regulators of myeloblastosis (*MYB*) genes [41], and miR166 family, regulators of Homeodomain-Leucine zipper (*HD*-*ZIP*) transcription factor families [42], were also lower than control sample during de-etiolation process in maize, but higher than control sample in rice (Additional file 8). On the contrary, miR528 family, regulators of *cupredoxin* domains involving oxidative stress responses [43], was expressed higher in maize than rice during de-etiolation process (Additional file 8). Similarly, miR827 family, verified regulators of *SPX* (named after *SYG1/PHO81/XPR1*)—major facilitator superfamily (*SPX*-*MFS*) genes [44], was expressed during de-etiolation in maize but not in rice (Additional file 8). Different from miRNA families discussed above, expression levels of miR164 family, reported regulators of the *CUP*-*SHAPED COTYLEDON* (*CUC*) genes [45], and miR390 family, regulators of lateral root development by cleaving *TRANS*-*ACTING SIRNA3* (*TAS3*) precursor RNA [46], converged to normal condition after 24-h illumination although different expression patterns between maize and rice were detected during de-etiolation process (Additional file 8, Fig. 4).

### mRNA targets and mimic lncRNA targets of miRNAs

The majority of plant miRNAs bind to its target mRNA through perfect reverse complementary matching [37]. A number of other factors also influence target binding, such as target accessibility, minimal free energy and secondary structure of miRNA-target pair. As a result, all these factors need to be considered when their binding targets are predicted [47]. In this study, we identified 47 and 42 candidate novel miRNAs for maize and rice respectively (Additional file 6). The mRNA targets of both known miRNAs and candidate novel miRNAs were predicted by psRNATarget [48] and listed in Additional file 9. We compared the obtained correlation coefficients between miRNAs and their binding targets. More rice miRNA-target pairs showed negative correlation coefficients than maize (Additional file 10). We further calculated the correlation coefficients between the expression patterns of miRNAs and their binding targets given by PTMED, a plant miRNA-target database based on microarray datasets [49]. Similar results were obtained, i.e., rice showed more negative correlations than maize.

Some of the correlation coefficients between expression values of miRNAs and their predicted targets were not negative. One possible explanation is that miRNAs can bind to not only mRNAs but also lncRNAs as target mimicry and thus affecting the miRNA-target regulation [50]. Given that a few previous studies have predicted genome-wide lncRNAs for maize [51, 52], here we tested whether those previously reported lncRNAs can serve as mimic targets of maize miRNAs (Additional file 11). In addition, by applying an in-house pipeline (see “Methods” section for details), we also identified 25 conserved lncRNAs precursors (13 from maize and 12 from rice genome). Two of the 12 predicted rice lncRNAs can potentially act as mimic target for two rice miRNAs (Additional file 12).

### The binding of miRNA to C_4_ photosynthesis related genes

To examine if miRNAs could be potentially involved with the regulation of photosynthesis, we examined the potential miRNAs that target photosynthesis related genes. We developed the list of photosynthesis related genes from a number of sources: (a) genes classified into photosynthesis category in MapMan annotation in maize and rice; (b) genes differentially expressed in bundle sheath cell and mesophyll cell in maize [53–55]; (c) rice orthologs of cell specific accumulated maize genes. miRNAs that predicted to have binding targets belonging to the list of photosynthesis related genes were listed in Table 1.

**Table 1:** Photosynthesis related genes as targets of identified miRNAs

miRNA	Target gene	Target loci	Cell	Annotation
*Z. mays*				
miR395	GRMZM2G051270	chr5:7702383:7702403:+	BS	Sulfate adenylyltransferase (*APS3*)
miR395	GRMZM2G149952	chr1:275091094:275091114:-	BS	Sulfate adenylyltransferase (*APS3*)
miR1432	GRMZM2G131489	chr7:81930813:81930833:+		Chlorophyll a/b binding family protein
miR528	GRMZM2G145101	chr7:127586688:127586708:-		Carbonic anhydrase family protein (*Beta*-*CA5*)
miR399	GRMZM2G146395	chr2:57541003:57541023:-		*FERREDOXIN 3* (*ATFD3*)
miR171	GRMZM5G800096	Pt:110960:110980:-		A plastid-encoded subunit of a NAD(P)H dehydrogenase complex (*NDHD*)
*O. sativa*				
miR395	LOC_Os03g53230	chr3:30532638:30532658:-	BS	Sulfate adenylyltransferase (*APS3*)
miR6251	LOC_Os03g53230	chr3:30533474:30533494:-	BS	Sulfate adenylyltransferase (*APS3*)
miR444	LOC_Os05g47560	chr5:27246682:27246702:-	M	Chloroplast serine-threonine protein kinase *STN7*
os_03_07	LOC_Os03g39610	chr3:22001370:22001390:+	M	Chlorophyll a/b binding family protein
miR393	LOC_Os08g09860	chr8:5693444:5693464:+		GLYCOLATE OXIDASE 1 (GOX1)
os_04_04	LOC_Os03g19380	chr3:10911399:10911419:+		*CP12* domain-containing protein
os_02_15	LOC_Os10g30550	chr10:15887085:15887105:-		Phosphoglycerate kinase
os_12_05	LOC_Os10g30550	chr10:15887086:15887106:-		Phosphoglycerate kinase

Photosynthesis gene list was composed of genes differentially expressed in bundle sheath (BS) and mesophyll (M) cell according to previous studies as well as genes under the “photosynthesis” category of MapMan annotation for maize and rice

Our anlaysis did not find any C_4_ cycle gene being a direct target of miRNA. However, a number of C_4_ photosynthesis related genes were predicted to be targets of miRNAs in both maize and rice. MiR395 has been reported to be crucial for sulfate homeostasis during growth and development in *Arabidopsis* [56]. Our data showed that miR395 regulates sulfate adenylytransferase (*APS3*) in both maize (GRMZM2G051270, GRMZM2G149952) and rice (LOC_Os03g53230), suggesting that this regulatory mechanism is conserved between maize and rice.

miR1432 was predicted to target calmodulin-binding protein (*CaMBP*) and EF-hand protein in rice [36]. Our data indicate that it might also target a chlorophyll a/b binding protein (GRMZM2G131489) in maize (Table 1). Chlorophyll a/b binding protein (LOC_Os03g39610) in rice was also predicted to be target of a candidate novel miRNA os_03_07 in rice. No sequence similarity was detected between miR1432 (CUCAGGAGAGAUGACACCGAC) and os_03_07 (AAUGACUUACAUUGUGGAACGGAG) mature sequences. This suggestes the mechanisms of chlorophyll a/b binding protein regulation might have been evolved through different routes between maize and rice.

*Beta*-*CA5* (GRMZM2G145101) was predicted to be a target of miR528 in maize. Notably this is not the *beta*-*CA* gene (GRMZM2G121878) that was recruited into C_4_ cycle in maize based on cell specific and leaf gradient data [53, 57]. miR528 was reported to regulate seed development in rice and response to drought/waterlogging stress in maize and rice [43, 58]. Thus the regulation of miR528 to *beta*-*CA* is more likely a response to environmental stress, in this case, a sudden exposure to light during de-etiolation process.

In addition, we also compared target gene lists of miRNAs with previously identified transcription factors, which might serve as putative positive or negative regulators of the development of Kranz anatomy [55]. Analysis showed that two target genes of miR156 (GRMZM2G097275 and GRMZM2G126018) were reported to be putative positive Kranz regulators [47].

### Experimental validation of predicted miRNA-target pairs

**Fig. 5:**
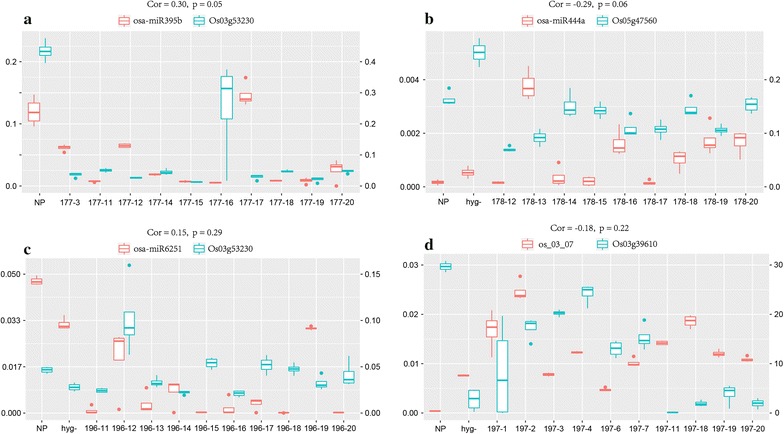
Experimental validation of four miRNA-target gene paris **a** osa-miR395b–Os03g53230 pair,  **b** osa-miR444a–Os05g47560 pair, **c** osa-miR6251–Os03g53230 pair, and **d** os_03_07–Os03g39610 pair. For each sub-plot, x axis represents different transgenic lines, NP and hyg- work as controls. *Left* y axis shows expression levels of miRNA and *right* y axis expression levels of corresponding target gene. Pearson correlation and *p* value were plotted as subtitle

**Fig. 6:**
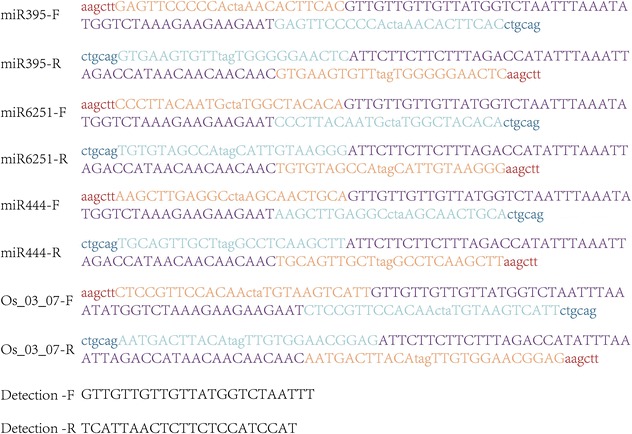
Short tandem target mimic sequences and primers. *Dark red* and *dark blue* nucleotides in lower-case marked *Hind*III site and Pstl site respectively. *Light red* and *light blue* nucleotides in upper-case represented miRNA sequence with a trinucleotide bulge. *Purple* nucleotides in the center worked as stem in the STMM

To validate our prediction of novel miRNAs and their regulation of target genes, we constructed short tandem targe mimic (STTM) vector sequences for four miRNA-target gene pairs, i.e., osa-miR395b–Os03g53230, osa-miR444a–Os05g47560, osa-miR6251–Os03g532230, Os_03_07–Os03g39610, and verified expression levels of both miRNAs and target genes among different transgenic lines in *O. sativa japonica* (Fig. 5). STTM sequence and detection primers were illustrated in Fig. 6.

Among four pairs we chose, two of them showed relative significant correlation between miRNA and predicted target gene with *p value* equals 0.05 and 0.06 indicating they might be ture miRNA-target pairs (Fig. 5a, b). miR444a, regulator of STN7, chloroplast serine-threonine protein kinase, showed negative correlation with its predicted target Os05g47560, whose orthologous gene in maize showed enriched expression in mesophyll cells in leaves (Fig. 5b; Table 1). While miR395b, regulator of sulfate adenylytransferase, showed positive correlation with its predicted target Os03g53230, whose maize ortholog showed enriched espression in bundle sheeth cell in leaves. Although the majority of known miRNAs repress gene expression by complementary mapping to their targets, miRNAs that positvely regulate target gene expression have been reported in other species as well [59, 60]. Our finding suggested this mechanism might also exist in rice.

Os_03_07, a novel miRNA predicted by our monocot specific pipeline, showed a negative correlation with its predicted target Os03g39610, a chlorophyll a/b binding protein (Fig. 5d). Other miRNA-target gene pairs predicted in this study as showed in Table 1 is still under experimental verfication.

## Discussion

### The de-etiolation system as a model to study regulation of C_4_ photosynthesis

We chose the greening process of the etiolated leaves for this study for the following seasons. First, light not only provides energy for photosynthesis, but also function as an environmental signal during development of photosynthesis [61]. Many photosynthetic genes showed altered expression during light induction [62] together with changes of many other genes, which makes it possible to use data collected during the de-etiolation process to predict miRNA-gene regulation pairs. During the de-etiolation process, we sampled 7 time points, i.e., 0, 0.5, 1, 3, 6, 12 and 24 h, following some previous studies [63–65]. Time interval was shorter during the beginning phase of de-etiolation process to better capture the initial responses of de-etiolated leaves to light illumination.

### Substantial level of conservation exists between maize and rice miRNAs

miRNAs serve as important regulators during plant growth and development [7]. There is substantial level of conservation in plant miRNAs. The existence of one of the most conserved miRNA family, miR166, was estimated to date back to at least 400 million years old [66]. More families of miRNAs were conserved in plants than animals [17]. This study showed a number of aspects of miRNAs are conserved between maize and rice. Firstly, maize and rice share 28 conserved miRNA families according to miRBase release 21 [19]. The majority of these families share not only similar mature sequences but also similar biological functions [58, 67, 68]. Many of them, i.e., miR156, miR160, miR164, miR166, miR167, miR171, miR172 and miR390, were suggested to play highly evolutionary conserved roles across plant species [54]. Secondly, in both maize and rice, the majority of plant miRNA targets are genes coding transcription factors rather than protein coding genes [5]. Thirdly, at least one member of 22 out of 28 conserved miRNA families were identified within maize and rice syntenic gene blocks, i.e., conserved blocks of order of genes within two sets of chromosomes that are being compared (Fig. 3), which suggested that they were inherited from the same ancestor.

For some miRNA families, their members form clusters together on the chromosome (Fig. 3). Though in both maize and rice, the majority of members of the same miRNA family scattered across different chromosomes, which is in contrast to animals where members of the same miRNA family tend to cluster together [69]. The fact that the same miRNA families of which their members form clusters on maize chromosomes also form clusters on rice chromosomes indicated the common evolutionary origins of these miRNA families between maize and rice (Fig. 3). In contrast, the scattered distributions of miRNA family members indicated that these members might have experienced fragment rearrangements possibly due to transposon activity during evolution.

### Diverse features between rice and maize miRNAs

Maize and rice miRNAs show many differences. Firstly, family sizes of maize miRNAs are much larger than those of rice miRNA families. On average, a maize miRNA family possesses about 11 members while a typical rice miRNA family only possesses about 2 members (miRBase release 21; Griffiths-Jones et al. [19]). The majority of identified maize miRNAs families are conserved across species while rice possess more species-specific miRNA families (miRBase release 21; Griffiths-Jones et al. [19]). Secondly, the average length of mature miRNA sequences is also 0.7-nt longer in rice than maize (Fig. 2). Rice possesses more 24-nt long miRNAs than maize (Fig. 2). Chen et al. [70] also reported a dominant portion of small RNAs of rice fall into 21-nt to 24-nt region, especially 24-nt. Montes et al. [71] reported that the majority of plant 24-nt sRNAs are heterochromatic siRNAs. Thus, one possible explanation might be some 24-nt siRNAs are mis-identified as miRNAs in rice as previous studies reported that 24-nt siRNAs are quite active in gene regulation in rice [72–74]. Given that the majority of plant miRNAs bind to targets by complementary reverse matching [37], much more targets were predicted in maize than in rice since maize miRNAs are usually shorter (Additional file 9). Thirdly, the correlations between miRNA expression profiles and target gene expression profiles are better in rice than maize (Additional file 10) suggesting a more sophisticated miRNA regulation network in rice. For those miRNAs that showed similar expression patterns between maize and rice, i.e., miR156, miR166, miR168, miR172, miR2275 and miR528, GO enrichment analysis of their predicted targets was applied (Additional file 13). Our analysis showed that their targets enriched in much less number of biological processes in maize compared to those in rice (Additional file 13), which also suggests that miRNAs might play more sophisticated regulatory roles in rice compared to maize.

It is worth mentioning that in the current miRBase, there are more rice miRNAs in miRBase than maize miRNAs, likely due to the larger number of publications on rice compared to maize. Interestingly, though a numbers of genome-wide identification of novel miRNAs have been conducted in maize [33–35, 75], the number of newly identified maize miRNAs did not dramatically increase the number of miRNAs. Thus, though maize genome (~2500 MB) is much larger than rice genome (~390 MB), maize might not necessarily possess more miRNAs than rice.

### Some miRNA families conserved between maize and rice showed drastically different expression patterns during de-etiolation

Although the majority of conserved miRNA families across species show similar functions [58, 67], their transcriptional responses during de-etiolation can be quite different. Here we used one example to illustrate this point. Earlier study showed that *HY5*, a global regulator of light responsive transcription factor, regulates expressions of eight miRNA genes, i.e., miR156d, miR172b, miR402, miR408, miR775, miR858, miR869 and miR1888, in *Arabidopsis* [25]. Among these 8 miRNAs, miR156, miR172 and miR408 are conserved miRNA families between maize and rice. Though miR156, regulators of flowering time, phase changing modulation, later embryonic maturation and root development [76], together with miR168, regulators of stress responses and signal transductions in plant development [67], showed very similar expression patterns between maize and rice during de-etiolation (Additional file 14). miR172, regulator of seed development and phase change in shoot [67], showed drastic difference in responses during de-etiolation, i.e., it was barely detectable in maize while constantly expressed in rice during de-etiolation (Additional file 8).

Dramatically different expresison patterns have also been detected in other miRNA families. miR156, miR160, miR164, miR166, miR167, miR171, miR172, and miR390, had been earlier reported to play evolutionarily conserved roles in plant development [54]. Though all these miRNA families share functions related to flowering control, embryonic maturation, root development, leaf primordial and development etc. [58, 67, 68], most of them showed different expression patterns upon exposure to light (Additional file 14) except (a) miR156, miR166, miR172, which showed almost identical expression curves, and (b) miR171 and miR390, which showed shifted expression patterns.

### Potential relevance of miRNAs and regulation of C_4_ photosynthesis

Given that the majority of miRNA targets are transcription factors genes that might further regulate gene expressions of other genes [5], it is rational to suggest that miRNAs, as upstream regulators, have the potential to function as master switches of some biological processes. Previous studies have reported that some miRNAs can move between different cells and cause cell specific accumulations [77], which raises the possibility that miRNAs might be related to cell specific expression of C_4_ enzymes. The large family size of plant miRNA families and relative frequent duplication events in plant genomes can potentially provide the conditions necessary for the evolution of neo-functions of some redundant miRNAs. Thus, we explored the predicted targets of both known and candidate novel miRNAs to investigate if miRNAs might be potentially associated with regulation of C_4_ photosynthesis.

Based on our results, no C_4_ cycle genes were identified to be direct target of known or predicted miRNAs (Additional file 9). However, a few photosynthesis related genes were predicted as targets of miRNAs (Table 1). *APS3* genes, i.e. GRMZM2G051270 and GRMZM2G149952 for maize and LOC_Os03g53230 for rice, were predicted to be binding targets of miR395 (Table 1). These two maize genes showed cell specific accumulations in maize according to Li et al. [45]. The similar regulation patterns of miR395 to *APS3* genes between maize and rice indicated that this regulatory mechanism might have existed long before the speciation of maize and rice, i.e. although *APS3* genes showed cell specific accumulation patterns in maize, which should not be direclty linked to C_4_ photosynthesis. *Beta*-*CA5* (GRMZM2G145101) was also predicted to be a binding target of miR528 in maize (Table 1). However, it is not the *beta*-*CA* gene (GRMZM2G121878) that was recruited into C_4_ photosynthesis in maize based on cell specific and leaf gradient data [53, 57]. Thus, these genes predicted to be targets of miRNAs are considered being peripheral to C_4_ photosynthesis. In fact, out of the total number of predicted miRNA binding targets, the photosynthesis related genes only represented a small fraction of them (Table 1). This indicates that miRNAs might have played a relatively minor role in the regulation of photosynthesis.

We further explored whether miRNAs might be involved in regulation of C_4_ photosynthesis by regulating C_4_ related transcription factors. We compared our target gene lists with putative positive or negative regulators of the development of Kranz anatomy reported by Wang et al. [47]. Our results showed that two target genes of miR156 (GRMZM2G097275 and GRMZM2G126018) were reported to be putative positive Kranz regulators (Additional file 15). Considering that miR156 is a highly conserved miRNA across plant species [76], we conducted GO enrichment anlaysis of all the predicted 24 and 21 target genes for maize and rice miR156 respectively. Analysis showed that the function of target gene lists is enriched with nucleus related functions in both species (Additional file 16). The functional significance of this miRNA and the corresponding binding targets in C_4_ Kranz anatomy formatoin needs to be tested next (Additional file 17).

## Conclusions

In this study, we examined the transcriptomic changes of miRNAs and their potential targets at 7 time points during the greening process of de-etiolated leaves in both maize and rice. No biological replicates were available in this study; instead we used 7 time points during the de-etiolation process to facilitate identification of miRNA-target gene pairs. We have parameterized a monocot specific prediction pipeline and identified 47 and 42 candidate novel miRNAs in maize and rice respectively. Further analysis showed that miRNA families are quite conserved between maize and rice in terms of mature sequence and genomic loci. However, there are also some different features between these miRNAs in two species such as the average length of mature sequence, family size and changes of expression profiles during de-etiolation. Our study further indicated that rice might possess a more complex miRNA regulation network than maize. Analysis of potential targets of both known and candidate novel miRNAs did not find any miRNA that direclty binds to C_4_ cycle genes. However indirect associations between miRNAs and C_4_ trait development related TFs were identified. Some of these identified miRNA and their binding targets need to be experimentally tested to examine their role during C_4_ photosynthesis development.

## Methods

### Plant materials, RNA isolations, miRNA and mRNA sequencing

*Zea mays L. ecotype B73* and *O. sativa japonica* seeds were sown and cultured in soil in dark condition at 28/22 °C and 60% humidity for 1 week. The 7-day-old etiolated seedlings were exposed to continuous light (~200 μmol/m^2^/s at the surface of the sampled leaves) and illuminated for 24 h. Seeds for control experiments were sown and cultured in soil for 1 week with a 16-h (7:00–23:00) light/8-h night cycle. Leaf sections of about 2 cm length were harvested from the third leaf at about top one-third of the position from tip to base. Samples were harvested before illumination (termed 0 h, at 9:00 a.m.) and six other time points after illumination, i.e., 0.5, 1, 3, 6, 12 and 24 h. These samples were immediately frozen in liquid nitrogen and stored at −80 °C until use. Total RNA was extracted with TRIzol^®^ protocol. The RNA integrity was evaluated by agarose gel electrophoresis and the concentration was checked using a Nanodrop ND-1000 spectrophotometer (Thermo Fisher Scientific, Wilmington, DE, USA). The qualified RNA samples were then taken to Illumina Sequencing Services of Beijing Genomics Institute (Shenzhen, China) where the cDNA libraries were built based on TruSeqTM Small RNA Sample Prep Kit (Illumina Inc, San Diego, CA) Illumina TruSeq™ RNA sample preparation v2 guide (Catalog # RS-122-2001) separately. For miRNA-seq, cDNA was further size-selected on agarose gels (145–160 bp) after the ligation of adapters. Sequencing was conducted by the Beijing Genomics Institute (Shenzhen, China) using Illumina HiSeq 2000 platform.

### Modification of miRDeep2 pipeline and prediction of novel miRNAs

A well-accepted miRNAs prediction package, miRDeep2 [32], was parameterized to be monocot specific. The altered parameters and scoring functions were described in our previous study [16]. The modified version of miRDeep2 is accessible via our website (http://www.picb.ac.cn/PSB/a/DOWNLOAD/). References miRNAs sequences required by miRDeep2 were downloaded from miRBase release 21, June 2014 (http://www.mirbase.org) [78].

The raw reads were poured into FASTX-toolkit pipeline version 0.0.13 (http://hannonlab.cshl.edu/fastx_toolkit/) to eliminate low-quality reads. Modified miRDeep2 pipeline was then applied to retained reads to remove 3′ adapter sequences and collapse redundant reads. Those collapsed reads were filtered against rRNA (http://www.arb-silva.de/no_cache/download/archive/release_111/Exports/), tRNA (http://gtrnadb.ucsc.edu/download.html) and snRNA (http://bioinf.scri.sari.ac.uk/cgi-bin/plant_snorna/home) databases to exclude other small RNAs from our analysis. Retained reads were then mapped against maize or rice genome (http://www.phytozome.net) to fetch potential miRNA precursor sequences, which was further examined by modified miRDeep2 pipeline. Predicted novel miRNAs were checked against known miRNAs across species for their conservation levels by using Gassst [79] (http://www.irisa.fr/symbiose/projects/gassst/).

We named candidate novel miRNAs by the combination of short forms of species names, chromosome number of precursor and order of candidate novel miRNAs discovered on that chromosome in this paper.

### miRNAs expression profiles, family characterization and genomic syntenies

Normalized TPQ (transcripts per quarter million) values were generated for both known and candidate novel miRNAs. Expression levels of miRNA families were determined by the sum of TPQ values of all family members. Differentially expressed miRNAs were detect by R package edgeR [80]. Expression curves were 3rd polynomial regressed to better illustrate time-dependent expression of miRNAs. This function has been used earlier to describe time-series dataset analysis [81, 82]. Maize and rice orthologous gene pairs were identified by BLASTX with maximum *e*-value as e-10. DAGchainer [83] was used to identify collinear regions amongst those orthologous genes. Two regions are considered to be collinear when at least five orthologous genes were found in the same order with no more than ten genes inserted between each neighbors. Family members and genomic syntenies of miRNAs were plotted by CIRCOS version 0.62 [84]. Two miRNA genes were considered syntenic if they belong to the same miRNA family and located within syntenic collinear regions.

### miRNA targets prediction, function analysis and lncRNA prediction

miRNA targets for known and candidate novel miRNAs were predicted by psRNATarget [48] (http://plantgrn.noble.org/psRNATarget/) using default parameters. GO enrichment analysis of the identified miRNA targets was performed using plantGSEA [85] (http://structuralbiology.cau.edu.cn/PlantGSEA/). Conserved lncRNAs were predicted by the following steps: (1) Identifying ortholog gene pairs that are syntenically conserved across *Z. mays*, *O. sativa* and *Setaria italica* by BBH-LS with default parameters [86]; (2) aligning the intergenic regions between ortholog gene pair with BLAST setting *e*-value cutoff to be 1e−6 [87]; (3) checking if any reads were mapped to this region by Bowtie2 version 2.2.6 [88]; (4) checking the secondary structure of expressed intergenic regions by RNAz 2.0 [89] with default parameters to identify precursor sequences of potential lncRNAs.

### Experimental validation of miRNA-target gene pairs

Short tandem target mimic modules (STTM) for each miRNA was annealed from a pair of complementary long primers which contained the spacer region and two same miRNA binding sites in, adjunct *Hind*III and *Pst*I sites were at the sides [90]. The miRNA binding sites include a CTA trinucleotide bulge corresponding to positions 10 and 11 from the 5′ end of the mature miRNAs, so that the STTM can effectively bind but will not be cleaved by the miRNAs. This module was then inserted between the 2X35S promoter and rbcS terminator of the binary vector pHB (12 kb) through the *Hind*III and *Pst*I sites. pHB uses the hygromycin gene and bar gene as selection markers. Single colonies were picked up for plasmid isolation with the detection primers and the constructs were verified by DNA sequencing. Transgenic plants were generated by *Agrobacterium tumefaciens*–mediated floral dip transformation. For each miRNA, about 10 transgened *O. sativa japonica* lines were obtained. RT-PCR was then perfomed for each individual plant to access the expression levels of both miRNAs and their corresponding target genes.

## Additional files

**Additional file 1.** Statistics of sequenced small RNA fragments across samples. The number of sequenced reads, retained reads after preprocessing, reads and tags that were aligned with other small RNAs were listed for all sequenced samples for both species.

**Additional file 2.** Numbers of reads and tags that aligned with other small RNA databases across samples. Dot sizes represent number of reads (A) and tags (B) of rRNAs, tRNAs, snRNAs and miRNAs as marked on the left panel. Abbreviation of species name and time after illumination in hours were listed on the top panel.

**Additional file 3.** Genomic loci of precursors of potential candidate novel miRNAs for maize and rice. These are all outputs without filtering from miRDeep2 pipeline parameterized with monocot specific parameters.

**Additional file 4.** Conservation of potential candidate novel miRNAs across species. The similarity cutoff was set to 90%. Known miRNA mature sequences were downloaded from miRBase release 21 [19].

**Additional file 5.** TPQ values of potential candidate novel miRNAs for maize and rice.

**Additional file 6.** Length, mature sequences, genomic loci of precursor sequence and TPQ values of candidate novel miRNAs. These are predictions filtered with expression criteria.

**Additional file 7.** Similarity between previously reported candidate novel miRNAs and miRNAs identified by this study. Candidate novel maize miRNAs predicted in our study were aligned to predictions of Wang et al. [24], Jiao et al. [25], Kang et al. [26] respectively. Candidate novel rice miRNAs was aligned to predictions of Sunkar et al. [27].

**Additional file 8.** Family-wise expression profiles of 28 conserved miRNA families in maize and rice. Expression level of a certain miRNA family was calculated by adding TPQ values of all members belonging to this family.

**Additional file 9.** Predicted target genes of both known and novel miRNAs for maize and rice. Potential targets for identified known and candidate novel miRNAs for both maize and rice were predicted by psRNATarget respectively. Aligned sequences and their genomic coordinates were also provided.

**Additional file 10.** Distribution of correlation coefficients between miRNAs and their predicted target genes. (A) Target gene listed obtained from PTMED database [41]. (B) Target gene lists predicted by psRNATarget.

**Additional file 11.** Potential mimic lncRNAs targets of known and novel maize miRNAs. Genome-wide lncRNAs in maize were predicted by Boerner et al. [43] and Li et al. [44]. MiRNA-lncRNA target pairs were identified by psRNATarget.

**Additional file 12.** Precursor sequences of predicted candidate lncRNAs and their potential to act as mimic targets for rice miRNAs. Predicted candidate lncRNA precursor sequences in maize and rice genomes were given in sheet 1 and 2 respectively. Sheet 3 listed two potential miRNA-mimic target relationship predicted by psRNATarget.

**Additional file 13.** GO enrichment analysis for targets of conserved miRNA families that showed similar expression patterns between maize and rice. Enriched GO labels were marked in bold.

**Additional file 14.** Expression patterns of 28 conserved miRNA families between maize and rice during de-etiolation. Curves were regression lines based on 3rd order polynomial regression and standard normalized from the original TPQ values.

**Additional file 15.** Potential mRNA targets of miRNAs that overlapped with Wang et al. [55] transcription factor lists

**Additional file 16.** GO enrichment analysis for targets of miR156 in maize and rice

**Additional file 17.** Expression patterns of conserved miRNAs and their predicted target genes. TPQ (transcripts per quarter million) values for all 7 time points were regressed using 3rd order polynomial regression. For *Zea mays* (A) and *Oryza sativa* (B), blue lines represent expression patterns of miRNAs. Grey lines represent expression patterns of their corresponding predicted target genes. Average values were plotted as red lines.

## Abbreviations

LncRNA: long non-coding RNA
miRNA: microRNA
TPQ: transcripts per quarter million
ARF8: auxin response factor 8
MYB: myeloblastosis
HD-ZIP: homeodomain Leucine zipper transcription factor
CUC: CUP-shaped cotyledon
TAS3: trans-acting siRNA3
PTMED: a plant miRNA target database
APS3: sulfate adenylyltransferase
CaMBP: Calmodulin-binding protein
CA5: carbonic anhydrase 5
STTM: short tandem target mimic
STN7: chloroplast serine threonine protein kinase

## References

[1] Dezulian T, Palatnik JF, Huson D, Weigel D (2005). Conservation and divergence of microRNA families in plants. Genome Biol..

[2] Unver T, Namuth-Covert DM, Budak H (2009). Review of current methodological approaches for characterizing microRNAs in plants. Int J Plant Genomics.

[3] Bartel DP, Lee R, Feinbaum R (2004). MicroRNAs: genomics, biogenesis, mechanism, and function genomics: the miRNA genes. Cell.

[4] Pritchard CC, Cheng HH, Tewari M. MicroRNA profiling: approaches and considerations. Nat Rev Genet. [Internet]. Nature Publishing Group; 2012 [cited 2014 Jan 9];13:358–69. http://www.ncbi.nlm.nih.gov/pubmed/22510765. 10.1038/nrg3198 PMC4517822 22510765

[5] Rhoades MW, Reinhart BJ, Lim LP, Burge CB, Bartel B, Bartel DP. Prediction of plant microRNA targets. Cell [Internet]. 2002;110:513–20. http://www.ncbi.nlm.nih.gov/pubmed/20430753. 10.1016/s0092-8674(02)00863-2 12202040

[6] Jones-Rhoades MW, Bartel DP, Bartel B. MicroRNAS and their regulatory roles in plants. Annu. Rev. Plant Biol. [Internet]. 2006 [cited 2013 Feb 27];57:19–53. http://www.ncbi.nlm.nih.gov/pubmed/16669754. 10.1146/annurev.arplant.57.032905.105218 16669754

[7] Eldem V, Okay S, Unver T (2013). Plant microRNAs: new players in functional genomics. Turk J Agric For.

[8] Ameres SL, Zamore PD. Diversifying microRNA sequence and function. Nat Rev Mol Cell Biol. [Internet]. Nature Publishing Group; 2013 [cited 2014 Jul 10];14:475–88. http://www.ncbi.nlm.nih.gov/pubmed/23800994. 10.1038/nrm3611 23800994

[9] Hu Z, Jiang Q, Ni Z, Chen R, Xu S, Zhang H. Analyses of a glycine max degradome library identify microRNA targets and microRNAs that trigger secondary siRNA biogenesis. J Integr Plant Biol. [Internet]. 2013 [cited 2014 Nov 19];55:160–76. doi:10.1111/jipb.12002. 10.1111/jipb.12002 23131131

[10] Zhao L, Lu X, Cao Y. MicroRNA and signal transduction pathways in tumor radiation response. Cell Signal [Internet]. Elsevier Inc.; 2013 [cited 2014 Nov 19];25:1625–34. http://linkinghub.elsevier.com/retrieve/pii/S0898656813001113. 10.1016/j.cellsig.2013.04.004 PMC3713775 23602933

[11] Moissiard G, Voinnet O. RNA silencing of host transcripts by cauliflower mosaic virus requires coordinated action of the four Arabidopsis Dicer-like proteins. PNAS [Internet]. 2006;103:19593–8. doi:10.1073/pnas.0604627103. 10.1073/pnas.0604627103 PMC1698440 17164336

[12] May P, Liao W, Wu Y, Shuai B, Richard McCombie W, Zhang MQ, et al. The effects of carbon dioxide and temperature on microRNA expression in Arabidopsis development. Nat Commun [Internet]. Nature Publishing Group, a division of Macmillan Publishers Limited. All Rights Reserved.; 2013;4:Article number 2145. doi:10.1038/ncomms3145. 10.1038/ncomms3145 23900278

[13] Zhang B, Pan X, Cobb GP, Anderson TA. Plant microRNA: a small regulatory molecule with big impact. Dev Biol. [Internet]. 2006 [cited 2013 Feb 27];289:3–16. http://www.ncbi.nlm.nih.gov/pubmed/16325172. 10.1016/j.ydbio.2005.10.036 16325172

[14] Baskerville S, Bartel DP (2005). Microarray profiling of microRNAs reveals frequent coexpression with neighboring miRNAs and host genes Microarray profiling of microRNAs reveals frequent coexpression with neighboring miRNAs and host genes. RNA.

[15] Naqvi AR, Sarwat M, Hasan S. Biogenesis, functions and fate of plant microRNAs. J Cell Physiol. 2012;3163–8. 10.1002/jcp.24052 22252306

[16] Thakur V, Wanchana S, Xu M, Bruskiewich R, Quick WP, Mosig A, et al. Characterization of statistical features for plant microRNA prediction. BMC Genomics [Internet]. BioMed Central Ltd; 2011 [cited 2011 Feb 17];12:108. http://www.biomedcentral.com/1471-2164/12/108. 10.1186/1471-2164-12-108 PMC3053258 21324149

[17] Sunkar R, Jagadeeswaran G. In silico identification of conserved microRNAs in large number of diverse plant species. BMC Plant Biol. [Internet]. 2008 [cited 2014 Apr 8];8:37. http://www.pubmedcentral.nih.gov/articlerender.fcgi?artid=2358906&tool=pmcentrez&rendertype=abstract. 10.1186/1471-2229-8-37 PMC2358906 18416839

[18] Zhang B, Pan X, Cannon CH, Cobb GP, Anderson TA. Conservation and divergence of plant microRNA genes. Plant J. [Internet]. 2006 [cited 2013 Feb 27];46:243–59. http://www.ncbi.nlm.nih.gov/pubmed/16623887. 10.1111/j.1365-313X.2006.02697.x 16623887

[19] Griffiths-Jones S, Grocock RJ, van Dongen S, Bateman A, Enright AJ. miRBase: microRNA sequences, targets and gene nomenclature. Nucleic Acids Res. [Internet]. 2006 [cited 2014 Mar 19];34:D140–4. http://www.pubmedcentral.nih.gov/articlerender.fcgi?artid=1347474&tool=pmcentrez&rendertype=abstract. 10.1093/nar/gkj112 PMC1347474 16381832

[20] Zhang S, Yue Y, Sheng L, Wu Y, Fan G, Li A, et al. PASmiR: a literature-curated database for miRNA molecular regulation in plant response to abiotic stress. BMC Plant Biol. [Internet]. 2013 [cited 2014 Nov 19];13:33. http://www.biomedcentral.com/1471-2229/13/33. 10.1186/1471-2229-13-33 PMC3599438 23448274

[21] Carnavale Bottino M, Rosario S, Grativol C, Thiebaut F, Rojas CA, Farrineli L, et al. High-throughput sequencing of small RNA transcriptome reveals salt stress regulated microRNAs in Sugarcane. Zhang J (ed). PLoS One [Internet]. 2013 [cited 2014 Nov 19];8:e59423. doi:10.1371/journal.pone.0059423. 10.1371/journal.pone.0059423 PMC3609749 23544066

[22] Sunkar R, Li Y-F, Jagadeeswaran G. Functions of microRNAs in plant stress responses. Trends Plant Sci. [Internet]. Elsevier Ltd; 2012 [cited 2014 Jan 14];17:196–203. http://www.ncbi.nlm.nih.gov/pubmed/22365280. 10.1016/j.tplants.2012.01.010 22365280

[23] Bauer D, Viczian A, Kircher S, Nobis T, Nitschke R, Kunkel T (2004). Constitutive photomorphogenesis 1 and multiple photoreceptors control degradation of phytochrome interacting factor 3, a transcription factor required for light signaling in Arabidopsis. Plant Cell.

[24] Martínez-García JF, Huq E, Quail PH. Direct targeting of light signals to a promoter element-bound transcription factor. Science (80) [Internet]. 2000 [cited 2014 Apr 16];288:859–63. doi:10.1126/science.288.5467.859. 10.1126/science.288.5467.859 10797009

[25] Zhang H, He H, Wang X, Wang X, Yang X, Li L, et al. Genome-wide mapping of the HY5-mediated gene networks in Arabidopsis that involve both transcriptional and post-transcriptional regulation. Plant J. [Internet]. 2011 [cited 2014 Mar 24];65:346–58. http://www.ncbi.nlm.nih.gov/pubmed/21265889. 10.1111/j.1365-313X.2010.04426.x 21265889

[26] Song Q-X, Liu Y-F, Hu X-Y, Zhang W-K, Ma B, Chen S-Y, et al. Identification of miRNAs and their target genes in developing soybean seeds by deep sequencing. BMC Plant Biol. [Internet]. BioMed Central Ltd; 2011 [cited 2014 Apr 10];11:5. http://www.pubmedcentral.nih.gov/articlerender.fcgi?artid=3023735&tool=pmcentrez&rendertype=abstract. 10.1186/1471-2229-11-5 PMC3023735 21219599

[27] Hibberd JM, Covshoff S. The regulation of gene expression required for C4 photosynthesis. Annu Rev Plant Biol. [Internet]. 2010 [cited 2012 Mar 9];61:181–207. http://www.ncbi.nlm.nih.gov/pubmed/20192753. 10.1146/annurev-arplant-042809-112238 20192753

[28] Williams BP, Aubry S, Hibberd JM. Molecular evolution of genes recruited into C4 photosynthesis. Trends Plant Sci. [Internet]. Elsevier Ltd; 2012 [cited 2012 Aug 27];17:213–20. http://www.ncbi.nlm.nih.gov/pubmed/22326564. 10.1016/j.tplants.2012.01.008 22326564

[29] Gowik U, Burscheidt J, Akyildiz M, Schlue U, Koczor M, Streubel M (2004). cis-regulatory elements for mesophyll-specific gene expression in the C4 plant Flaveria trinervia, the promoter of the C4 phosphoenolpyruvate carboxylase gene. Plant Cell.

[30] Wang L, Peterson RB, Brutnell TP. Regulatory mechanisms underlying C4 photosynthesis. New Phytol. [Internet]. 2011 [cited 2011 Feb 14];190:9–20. http://www.ncbi.nlm.nih.gov/pubmed/21299565. 10.1111/j.1469-8137.2011.03649.x 21299565

[31] Kim VN. MicroRNA biogenesis: coordinated cropping and dicing. Nat Rev Mol Cell Biol. [Internet]. 2005 [cited 2011 Jul 9];6:376–85. http://www.ncbi.nlm.nih.gov/pubmed/15852042. 10.1038/nrm1644 15852042

[32] Friedländer MR, Mackowiak SD, Li N, Chen W, Rajewsky N. miRDeep2 accurately identifies known and hundreds of novel microRNA genes in seven animal clades. Nucleic Acids Res. [Internet]. 2012 [cited 2013 Mar 9];40:37–52. http://www.pubmedcentral.nih.gov/articlerender.fcgi?artid=3245920&tool=pmcentrez&rendertype=abstract. 10.1093/nar/gkr688 PMC3245920 21911355

[33] Wang L, Liu H, Li D, Chen H. Identification and characterization of maize microRNAs involved in the very early stage of seed germination. BMC Genomics [Internet]. BioMed Central Ltd; 2011 [cited 2014 Apr 4];12:154. http://www.pubmedcentral.nih.gov/articlerender.fcgi?artid=3066126&tool=pmcentrez&rendertype=abstract. 10.1186/1471-2164-12-154 PMC3066126 21414237

[34] Jiao Y, Song W, Zhang M, Lai J. Identification of novel maize miRNAs by measuring the precision of precursor processing. BMC Plant Biol. [Internet]. BioMed Central Ltd; 2011 [cited 2014 Apr 4];11:141. http://www.pubmedcentral.nih.gov/articlerender.fcgi?artid=3214924&tool=pmcentrez&rendertype=abstract. 10.1186/1471-2229-11-141 PMC3214924 22014170

[35] Kang M, Zhao Q, Zhu D, Yu J. Characterization of microRNAs expression during maize seed development. BMC Genomics [Internet]. 2012;13:360. http://www.pubmedcentral.nih.gov/articlerender.fcgi?artid=3468377&tool=pmcentrez&rendertype=abstract. 10.1186/1471-2164-13-360 PMC3468377 22853295

[36] Sunkar R, Zhou X, Zheng Y, Zhang W, Zhu J-K. Identification of novel and candidate miRNAs in rice by high throughput sequencing. BMC Plant Biol. [Internet]. 2008 [cited 2014 Feb 26];8:25. http://www.pubmedcentral.nih.gov/articlerender.fcgi?artid=2292181&tool=pmcentrez&rendertype=abstract. 10.1186/1471-2229-8-25 PMC2292181 18312648

[37] Carthew RW, Sontheimer EJ. Origins and mechanisms of miRNAs and siRNAs. Cell [Internet]. Elsevier Inc.; 2009 [cited 2013 Feb 27];136:642–55. http://www.pubmedcentral.nih.gov/articlerender.fcgi?artid=2675692&tool=pmcentrez&rendertype=abstract. 10.1016/j.cell.2009.01.035 PMC2675692 19239886

[38] Li A, Mao L. Evolution of plant microRNA gene families. Cell Res. [Internet]. 2007 [cited 2013 Mar 11];17:212–8. http://www.ncbi.nlm.nih.gov/pubmed/17130846. 10.1038/sj.cr.7310113 17130846

[39] Pashkovskiy PP, Ryazansky SS (2013). Biogenesis, evolution, and functions of plant microRNAs. Biochemistry.

[40] Yang JH, Han SJ, Yoon EK, Lee WS. Evidence of an auxin signal pathway, microRNA167-ARF8-GH3, and its response to exogenous auxin in cultured rice cells. Nucleic Acids Res. [Internet]. 2006 [cited 2013 Mar 20];34:1892–9. http://www.pubmedcentral.nih.gov/articlerender.fcgi?artid=1447648&tool=pmcentrez&rendertype=abstract. 10.1093/nar/gkl118 PMC1447648 16598073

[41] Axtell MJ. Classification and comparison of small RNAs from plants. Annu Rev Plant Biol. [Internet]. 2013 [cited 2013 Mar 1];5.1–5.23. http://www.ncbi.nlm.nih.gov/pubmed/23330790. 10.1146/annurev-arplant-050312-120043 23330790

[42] Rubio-Somoza I, Weigel D. MicroRNA networks and developmental plasticity in plants. Trends Plant Sci. [Internet]. Elsevier Ltd; 2011 [cited 2013 Mar 7];16:258–64. http://www.ncbi.nlm.nih.gov/pubmed/21466971. 10.1016/j.tplants.2011.03.001 21466971

[43] Liu Z, Kumari S, Zhang L, Zheng Y, Ware D. Characterization of miRNAs in response to short-term waterlogging in three inbred lines of Zea mays. PLoS One [Internet]. 2012 [cited 2014 Feb 26];7:e39786. http://www.pubmedcentral.nih.gov/articlerender.fcgi?artid=3387268&tool=pmcentrez&rendertype=abstract. 10.1371/journal.pone.0039786 PMC3387268 22768123

[44] Lin S-I, Santi C, Jobet E, Lacut E, El Kholti N, Karlowski WM, et al. Complex regulation of two target genes encoding SPX-MFS proteins by rice miR827 in response to phosphate starvation. Plant Cell Physiol. [Internet]. 2010 [cited 2013 Mar 7];51:2119–31. http://www.ncbi.nlm.nih.gov/pubmed/21062869. 10.1093/pcp/pcq170 21062869

[45] Sieber P, Wellmer F, Gheyselinck J, Riechmann JL, Meyerowitz EM. Redundancy and specialization among plant microRNAs: role of the MIR164 family in developmental robustness. Development [Internet]. 2007 [cited 2013 Mar 11];134:1051–60. http://www.ncbi.nlm.nih.gov/pubmed/17287247. 10.1242/dev.02817 17287247

[46] Yoon EK, Yang JH, Lim J, Kim SH, Kim S-K, Lee WS. Auxin regulation of the microRNA390-dependent transacting small interfering RNA pathway in Arabidopsis lateral root development. Nucleic Acids Res. [Internet]. 2010 [cited 2014 Apr 28];38:1382–91. http://www.pubmedcentral.nih.gov/articlerender.fcgi?artid=2831332&tool=pmcentrez&rendertype=abstract. 10.1093/nar/gkp1128 PMC2831332 19969544

[47] Dai X, Zhuang Z, Zhao PX. Computational analysis of miRNA targets in plants: current status and challenges. Brief Bioinform. [Internet]. 2011 [cited 2014 Jan 16];12:115–21. http://www.ncbi.nlm.nih.gov/pubmed/20858738. 10.1093/bib/bbq065 20858738

[48] Dai X, Zhao PX. psRNATarget: a plant small RNA target analysis server. Nucleic Acids Res. [Internet]. 2011 [cited 2014 Jan 24];39:W155–9. http://www.pubmedcentral.nih.gov/articlerender.fcgi?artid=3125753&tool=pmcentrez&rendertype=abstract. 10.1093/nar/gkr319 PMC3125753 21622958

[49] Sun X, Dong B, Yin L, Zhang R, Du W, Liu D, et al. PMTED: a plant microRNA target expression database. BMC Bioinform. [Internet]. BMC Bioinformatics; 2013 [cited 2014 Jan 16];14:174. http://www.pubmedcentral.nih.gov/articlerender.fcgi?artid=3680227&tool=pmcentrez&rendertype=abstract. 10.1186/1471-2105-14-174 PMC3680227 23725466

[50] Wu H-J, Wang Z-M, Wang M, Wang X-J. Widespread long noncoding RNAs as endogenous target mimics for microRNAs in plants. Plant Physiol. [Internet]. 2013 [cited 2014 Jan 16];161:1875–84. http://www.ncbi.nlm.nih.gov/pubmed/23429259. 10.1104/pp.113.215962 PMC3613462 23429259

[51] Boerner S, McGinnis KM. Computational identification and functional predictions of long noncoding RNA in Zea mays. PLoS One [Internet]. 2012 [cited 2014 Mar 21];7:e43047. http://www.pubmedcentral.nih.gov/articlerender.fcgi?artid=3420876&tool=pmcentrez&rendertype=abstract. 10.1371/journal.pone.0043047 PMC3420876 22916204

[52] Li L, Eichten SR, Shimizu R, Petsch K, Yeh C-T, Wu W, et al. Genome-wide discovery and characterization of maize long non-coding RNAs. Genome Biol. [Internet]. 2014 [cited 2014 Mar 20];15:R40. http://genomebiology.com/2014/15/2/R40. 10.1186/gb-2014-15-2-r40 PMC4053991 24576388

[53] Li P, Ponnala L, Gandotra N, Wang L, Si Y, Tausta SL, et al. The developmental dynamics of the maize leaf transcriptome. Nat Genet. [Internet]. Nature Publishing Group; 2010 [cited 2010 Nov 4];42:1060–7. http://www.ncbi.nlm.nih.gov/pubmed/21037569. 10.1038/ng.703 21037569

[54] Chang Y-M, Liu W-Y, Shih a. C-C, Shen M-N, Lu C-H, Lu M-YJ, et al. Characterizing regulatory and functional differentiation between maize mesophyll and bundle sheath cells by transcriptomic analysis. Plant Physiol. [Internet]. 2012 [cited 2012 Jul 25];160:165–77. http://www.plantphysiol.org/cgi/doi/10.1104/pp.112.203810. 10.1104/pp.112.203810 PMC3440195 22829318

[55] Wang P, Kelly S, Fouracre JP, Langdale J a. Genome-wide transcript analysis of early maize leaf development reveals gene cohorts associated with the differentiation of C4 Kranz anatomy. Plant J. [Internet]. 2013 [cited 2014 Jan 29];75:656–70. http://www.ncbi.nlm.nih.gov/pubmed/23647263. 10.1111/tpj.12229 23647263

[56] Liang G, Yu D. Reciprocal regulation among miR395, APS and SULTR2;1 in Arabidopsis thaliana. Plant Signal Behav. [Internet]. 2010 [cited 2014 Feb 26];5:1257–9. http://www.landesbioscience.com/journals/psb/article/12608/. 10.4161/psb.5.10.12608 PMC3115361 20935495

[57] Pick TR, Bräutigam A, Schlüter U, Denton AK, Colmsee C, Scholz U, et al. Systems analysis of a maize leaf developmental gradient redefines the current C4 model and provides candidates for regulation. Plant Cell [Internet]. 2011 [cited 2014 Mar 20];23:4208–20. http://www.pubmedcentral.nih.gov/articlerender.fcgi?artid=3269860&tool=pmcentrez&rendertype=abstract. 10.1105/tpc.111.090324 PMC3269860 22186372

[58] Zhou L, Liu Y, Liu Z, Kong D, Duan M, Luo L. Genome-wide identification and analysis of drought-responsive microRNAs in Oryza sativa. J Exp Bot. [Internet]. 2010 [cited 2014 Jan 11];61:4157–68. http://www.ncbi.nlm.nih.gov/pubmed/20729483. 10.1093/jxb/erq237 20729483

[59] Chatterjee S, Fasler M, Bu I. Short article target-mediated protection of endogenous MicroRNAs in *C. elegans*. 2011;7:388–96. 10.1016/j.devcel.2011.02.008 21397849

[60] Pasquinelli AE. MicroRNAs and their targets : recognition, regulation and an emerging reciprocal relationship. Nat Rev Genet. [Internet]. Nature Publishing Group; 2012;13:271–82. http://dx.doi.org/10.1038/nrg3162. 10.1038/nrg3162 22411466

[61] Deng XW, Quail PH. Signalling in light-controlled development. Semin Cell Dev Biol. [Internet]. 1999;10:121–9. http://www.ncbi.nlm.nih.gov/pubmed/10441064. 10.1006/scdb.1999.0287 10441064

[62] Shen Z, Li P, Ni R-J, Ritchie M, Yang C-P, Liu G-JG-F, et al. Label-free quantitative proteomics analysis of etiolated maize seedling leaves during greening. Mol Cell Proteomics. [Internet]. 2009 [cited 2011 Jan 13];8:2443–60. http://www.pubmedcentral.nih.gov/articlerender.fcgi?artid=2773713&tool=pmcentrez&rendertype=abstract. 10.1074/mcp.M900187-MCP200 PMC2773713 19666873

[63] Schaffner AR, Sheen J (1991). Maize rbcS promoter activity depends on sequence elements not found in dicot. Plant Cell.

[64] Sheen J. Molecular mechanisms underlying the differential expression of maize pyruvate, orthophosphate dikinase genes. Plant Cell. [Internet]. 1991;3:225–45. http://www.pubmedcentral.nih.gov/articlerender.fcgi?artid=159995&tool=pmcentrez&rendertype=abstract. 10.1105/tpc.3.3.225 PMC159995 1668653

[65] Kausch a P, Owen TP, Zachwieja SJ, Flynn AR, Sheen J. Mesophyll-specific, light and metabolic regulation of the C4 PPCZm1 promoter in transgenic maize. Plant Mol Biol. [Internet]. 2001;45:1–15. http://www.ncbi.nlm.nih.gov/pubmed/11247600. 10.1023/a:1006487326533 11247600

[66] Floyd SK, Bowman JL. Ancient microRNA target sequences in plants. Nature [Internet]. 2004 [cited 2014 Mar 21];428:1 p following 486. http://www.ncbi.nlm.nih.gov/pubmed/15058298. 10.1038/428485a 15057819

[67] Jin D, Wang Y, Zhao Y, Chen M. MicroRNAs and their cross-talks in plant development. J Genet Genomics [Internet]. Elsevier Limited and Science Press; 2013 [cited 2014 Feb 18];40:161–70. http://www.ncbi.nlm.nih.gov/pubmed/23618399. 10.1016/j.jgg.2013.02.003 23618399

[68] Zeng H, Wang G, Hu X, Wang H, Du L, Zhu Y. Role of microRNAs in plant responses to nutrient stress. Plant Soil [Internet]. 2013 [cited 2014 Jan 16];374:1005–21. http://link.springer.com/10.1007/s11104-013-1907-6.

[69] Guddeti S, Zhang DC, Li AL, Leseberg CH, Kang H, Li XG, et al. Molecular evolution of the rice miR395 gene family. Cell Res. [Internet]. 2005;15:631–8. http://www.ncbi.nlm.nih.gov/pubmed/16117853. 10.1038/sj.cr.7290333 16117853

[70] Chen M, Meng Y, Gu H, Chen D. Functional characterization of plant small RNAs based on next-generation sequencing data. Comput Biol Chem. [Internet]. Elsevier Ltd; 2010 [cited 2013 Mar 11];34:308–12. http://www.ncbi.nlm.nih.gov/pubmed/21030312. 10.1016/j.compbiolchem.2010.10.001 21030312

[71] Montes RAC, de Fátima Rosas-Cárdenas F, De Paoli E, Accerbi M, Rymarquis LA, Mahalingam G (2014). Sample sequencing of vascular plants demonstrates widespread conservation and divergence of microRNAs. Nat Commun.

[72] Henderson IR, Jacobsen SE. Epigenetic inheritance in plants. Nature [Internet]. 2007 [cited 2014 Mar 22];447:418–24. http://www.ncbi.nlm.nih.gov/pubmed/17522675. 10.1038/nature05917 17522675

[73] Qi Y, He X, Wang X-J, Kohany O, Jurka J, Hannon GJ. Distinct catalytic and non-catalytic roles of ARGONAUTE4 in RNA-directed DNA methylation. Nature [Internet]. 2006 [cited 2014 Mar 20];443:1008–12. http://www.ncbi.nlm.nih.gov/pubmed/16998468. 10.1038/nature05198 16998468

[74] Zilberman D, Gehring M, Tran RK, Ballinger T, Henikoff S. Genome-wide analysis of Arabidopsis thaliana DNA methylation uncovers an interdependence between methylation and transcription. Nat Genet. [Internet]. 2007 [cited 2014 Mar 20];39:61–9. http://www.ncbi.nlm.nih.gov/pubmed/17128275. 10.1038/ng1929 17128275

[75] Zhang L, Chia J-M, Kumari S, Stein JC, Liu Z, Narechania A, et al. A genome-wide characterization of microRNA genes in maize. PLoS Genet. [Internet]. 2009 [cited 2013 Mar 11];5:e1000716. http://www.pubmedcentral.nih.gov/articlerender.fcgi?artid=2773440&tool=pmcentrez&rendertype=abstract. 10.1371/journal.pgen.1000716 PMC2773440 19936050

[76] Xie K, Shen J, Hou X, Yao J, Li X, Xiao J, et al. Gradual increase of miR156 regulates temporal expression changes of numerous genes during leaf development in rice. Plant Physiol. [Internet]. 2012 [cited 2013 Mar 11];158:1382–94. http://www.pubmedcentral.nih.gov/articlerender.fcgi?artid=3291253&tool=pmcentrez&rendertype=abstract. 10.1104/pp.111.190488 PMC3291253 22271747

[77] Chitwood DH, Timmermans MCP. Small RNAs are on the move. Nature [Internet]. Nature Publishing Group; 2010 [cited 2014 Feb 25];467:415–9. http://www.ncbi.nlm.nih.gov/pubmed/20864994. 10.1038/nature09351 20864994

[78] Kozomara A, Griffiths-Jones S. miRBase: integrating microRNA annotation and deep-sequencing data. Nucleic Acids Res. [Internet]. 2011 [cited 2014 Jan 9];39:D152–7. http://www.pubmedcentral.nih.gov/articlerender.fcgi?artid=3013655&tool=pmcentrez&rendertype=abstract. 10.1093/nar/gkq1027 PMC3013655 21037258

[79] Rizk G, Lavenier D. GASSST: global alignment short sequence search tool. Bioinformatics [Internet]. 2010 [cited 2013 Mar 11];26:2534–40. http://www.pubmedcentral.nih.gov/articlerender.fcgi?artid=2951093&tool=pmcentrez&rendertype=abstract. 10.1093/bioinformatics/btq485 PMC2951093 20739310

[80] Robinson MD, McCarthy DJ, Smyth GK. edgeR: a Bioconductor package for differential expression analysis of digital gene expression data. Bioinformatics [Internet]. 2010 [cited 2014 Apr 28];26:139–40. http://www.pubmedcentral.nih.gov/articlerender.fcgi?artid=2796818&tool=pmcentrez&rendertype=abstract. 10.1093/bioinformatics/btp616 PMC2796818 19910308

[81] Masry E (1996). Multivariate local polynomial regression for time series: uniform strong consistency and rates. J Time Ser Anal.

[82] Chen Y, Dong G, Han J, Wah BW, Wang J. Multi-dimensional regression analysis of time-series data streams, in *Proceedings of the 28th International Conference Very Large Data Bases*. 2002;323–34.

[83] Haas BJ, Delcher AL, Wortman JR, Salzberg SL. DAGchainer: a tool for mining segmental genome duplications and synteny. Bioinformatics [Internet]. 2004 [cited 2012 Mar 20];20:3643–6. http://www.ncbi.nlm.nih.gov/pubmed/15247098. 10.1093/bioinformatics/bth397 15247098

[84] Krzywinski M, Schein J, Birol I, Connors J, Gascoyne R, Horsman D, et al. Circos: an information aesthetic for comparative genomics. Genome Res. [Internet]. 2009 [cited 2012 Jul 13];19:1639–45. http://www.pubmedcentral.nih.gov/articlerender.fcgi?artid=2752132&tool=pmcentrez&rendertype=abstract. 10.1101/gr.092759.109 PMC2752132 19541911

[85] Yi X, Du Z, Su Z. PlantGSEA: a gene set enrichment analysis toolkit for plant community. Nucleic Acids Res. [Internet]. 2013 [cited 2014 Mar 4];41:W98–103. http://www.pubmedcentral.nih.gov/articlerender.fcgi?artid=3692080&tool=pmcentrez&rendertype=abstract. 10.1093/nar/gkt281 PMC3692080 23632162

[86] Zhang M, Leong HW. BBH-LS: an algorithm for computing positional homologs using sequence and gene context similarity. BMC Syst Biol. [Internet]. BioMed Central Ltd; 2012 [cited 2013 Jun 7];6(Suppl 1):S22. http://www.pubmedcentral.nih.gov/articlerender.fcgi?artid=3403649&tool=pmcentrez&rendertype=abstract. 10.1186/1752-0509-6-S1-S22 PMC3403649 23046607

[87] Altschup SF, Gish W, Pennsylvania T, Park U (1990). Basic local alignment search tool. J Mol Biol.

[88] Langmead B, Salzberg SL. Fast gapped-read alignment with Bowtie 2. Nat Methods [Internet]. 2012 [cited 2012 Jul 16];9:357–9. http://www.ncbi.nlm.nih.gov/pubmed/22388286. 10.1038/nmeth.1923 PMC3322381 22388286

[89] Gruber AR, Findeiß S, Washietl S, Hofacker IL, Stadler PF. RNAz 2.0: improved noncoding RNA detection. Pac Symp Biocomput. [Internet]. 2010;69–79. http://www.ncbi.nlm.nih.gov/pubmed/19908359. 19908359

[90] Yan J, Gu Y, Jia X, Kang W, Pan S, Tang X (2012). Effective Small RNA Destruction by the Expression of a Short Tandem Target Mimic in Arabidopsis. Plant Cell.

