# Supplementary figures and images for: Altered expression profiles of microRNA families during de-etiolation of maize and rice leaves
Source: BMC Res Notes. 2017 Feb 24;10:108. doi: 10.1186/s13104-016-2367-x (PMC5324284; doi:10.1186/s13104-016-2367-x)

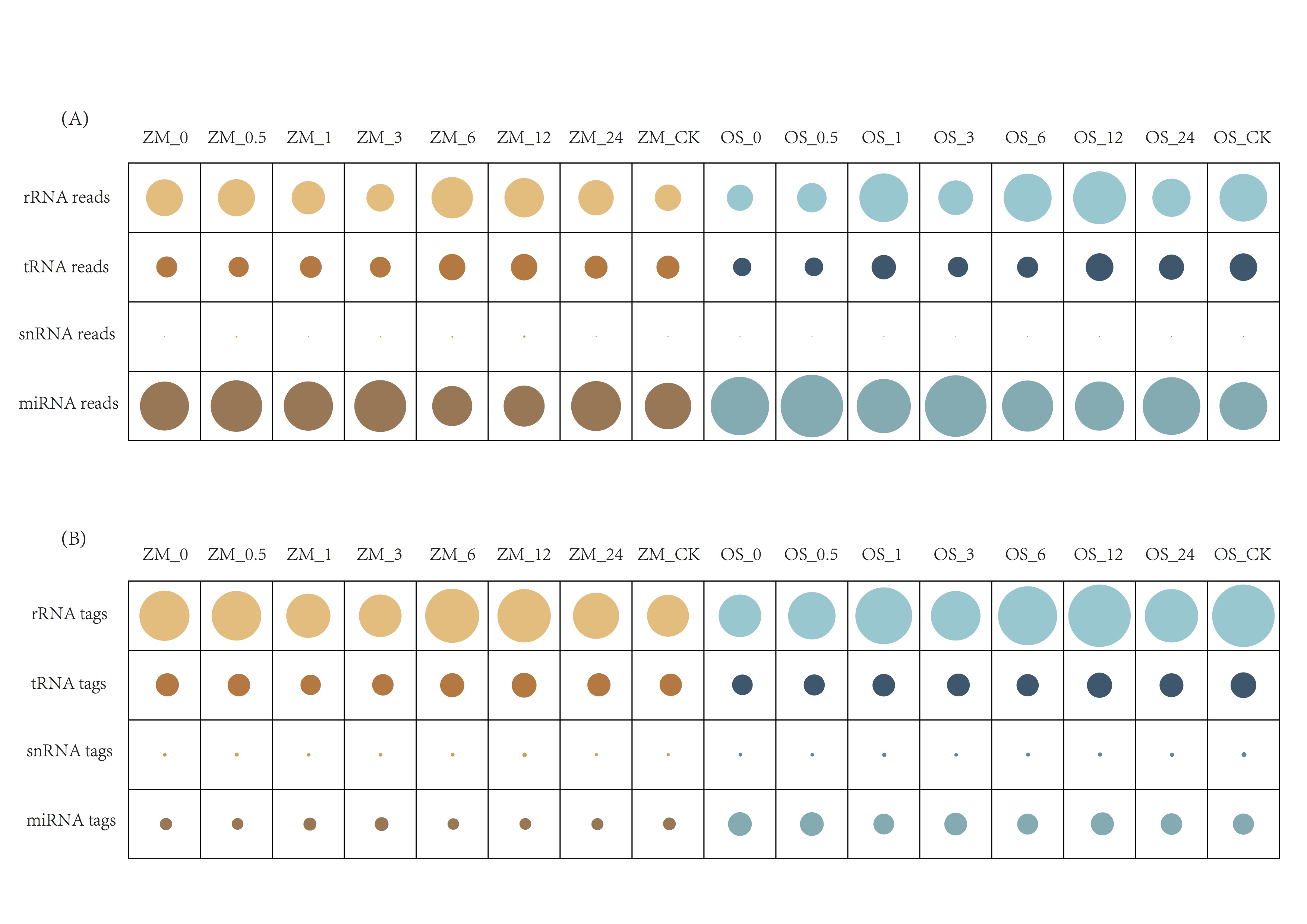

Supplement: Supplementary file 2 — Additional file 2. Numbers of reads and tags that aligned with other small RNA databases across samples. Dot sizes represent number of reads (A) and tags (B) of rRNAs, tRNAs, snRNAs and miRNAs as marked on the left panel. Abbreviation of species name and time after illumination in hours were listed on the top panel. [file 13104_2016_2367_MOESM2_ESM.jpg]

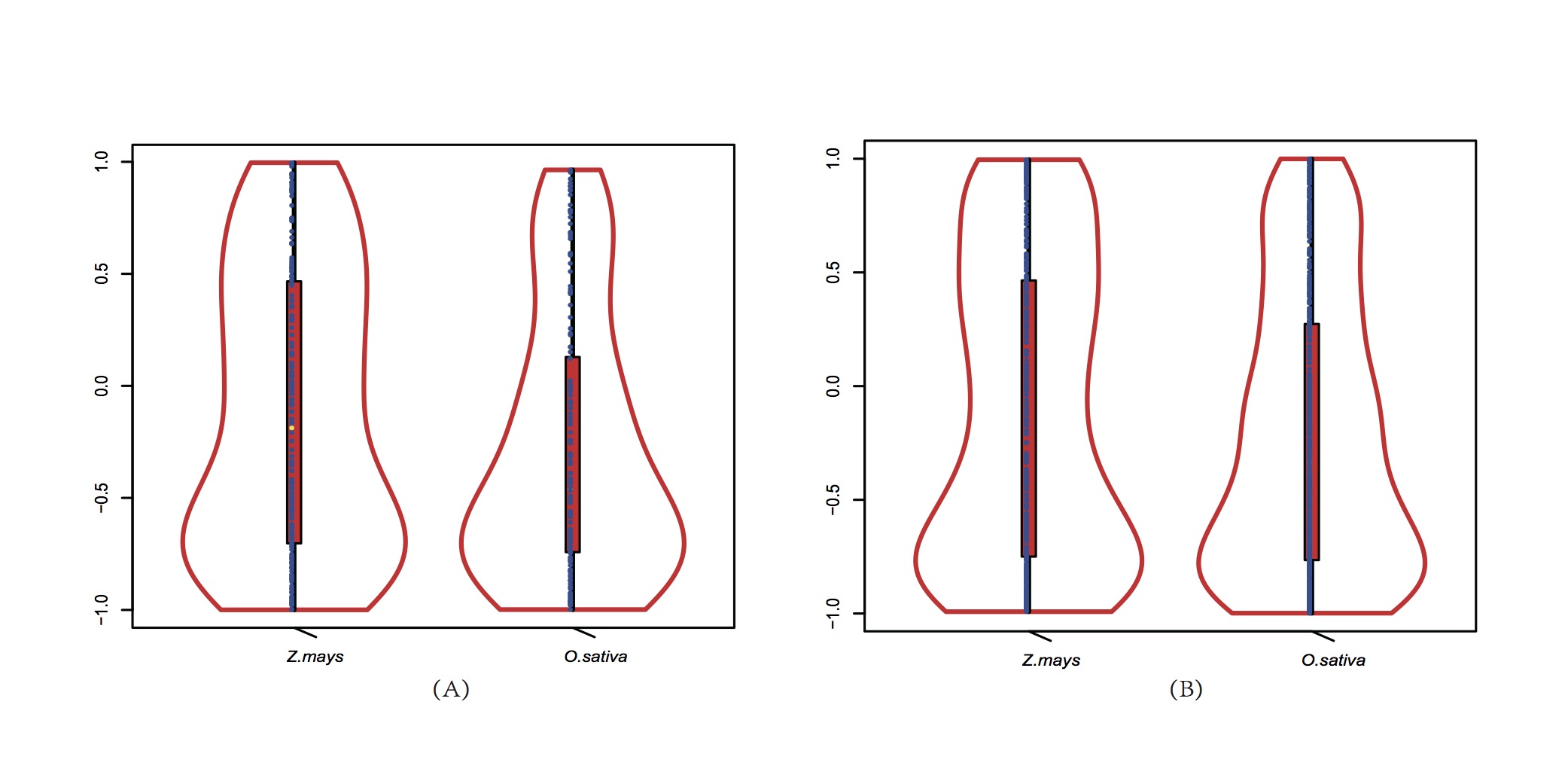

Supplement: Supplementary file 10 — Additional file 10. Distribution of correlation coefficients between miRNAs and their predicted target genes. (A) Target gene listed obtained from PTMED database [41]. (B) Target gene lists predicted by psRNATarget. [file 13104_2016_2367_MOESM10_ESM.jpg]

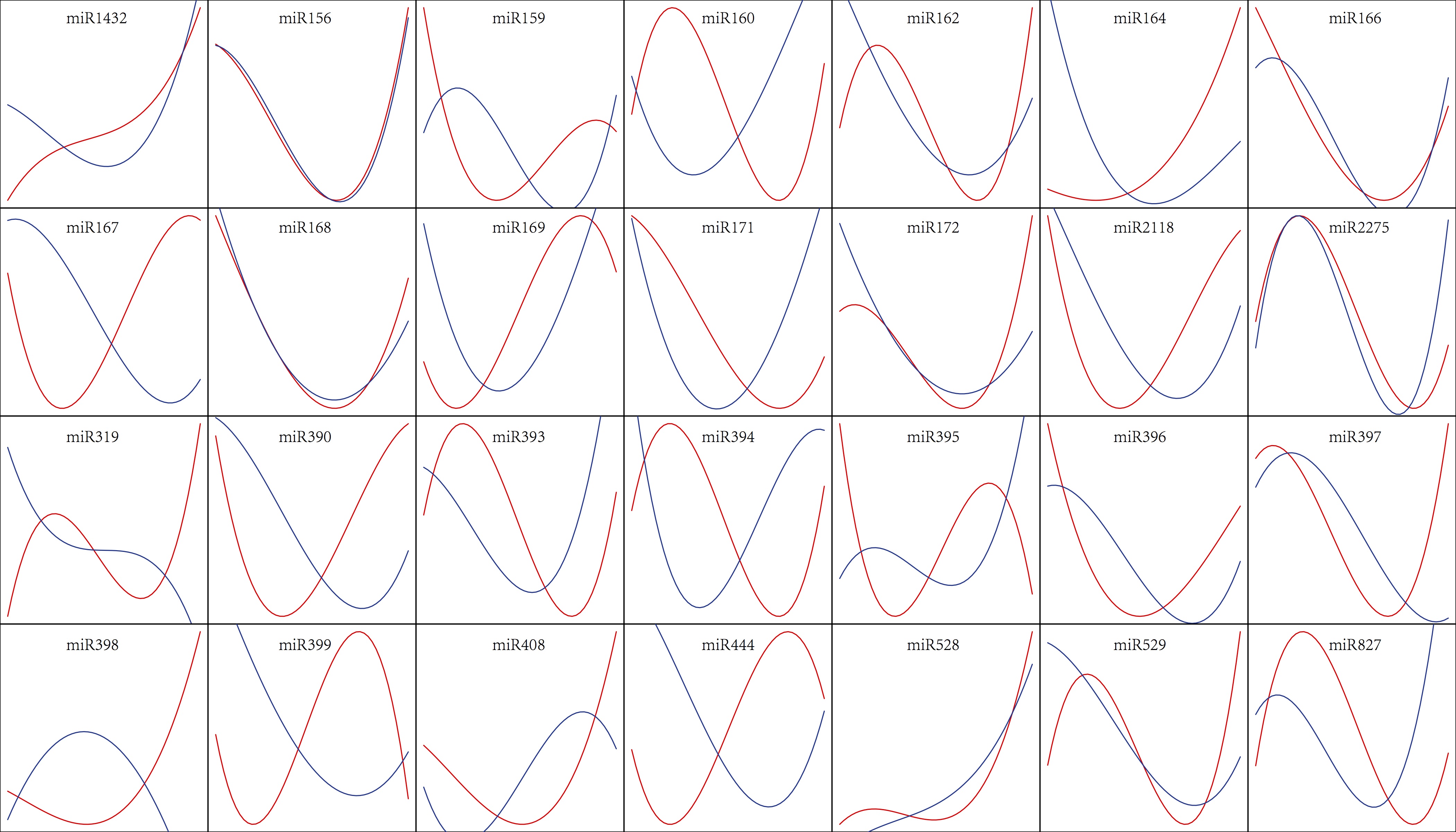

Supplement: Supplementary file 14 — Additional file 14. Expression patterns of 28 conserved miRNA families between maize and rice during de-etiolation. Curves were regression lines based on 3rd order polynomial regression and standard normalized from the original TPQ values. [file 13104_2016_2367_MOESM14_ESM.jpg]

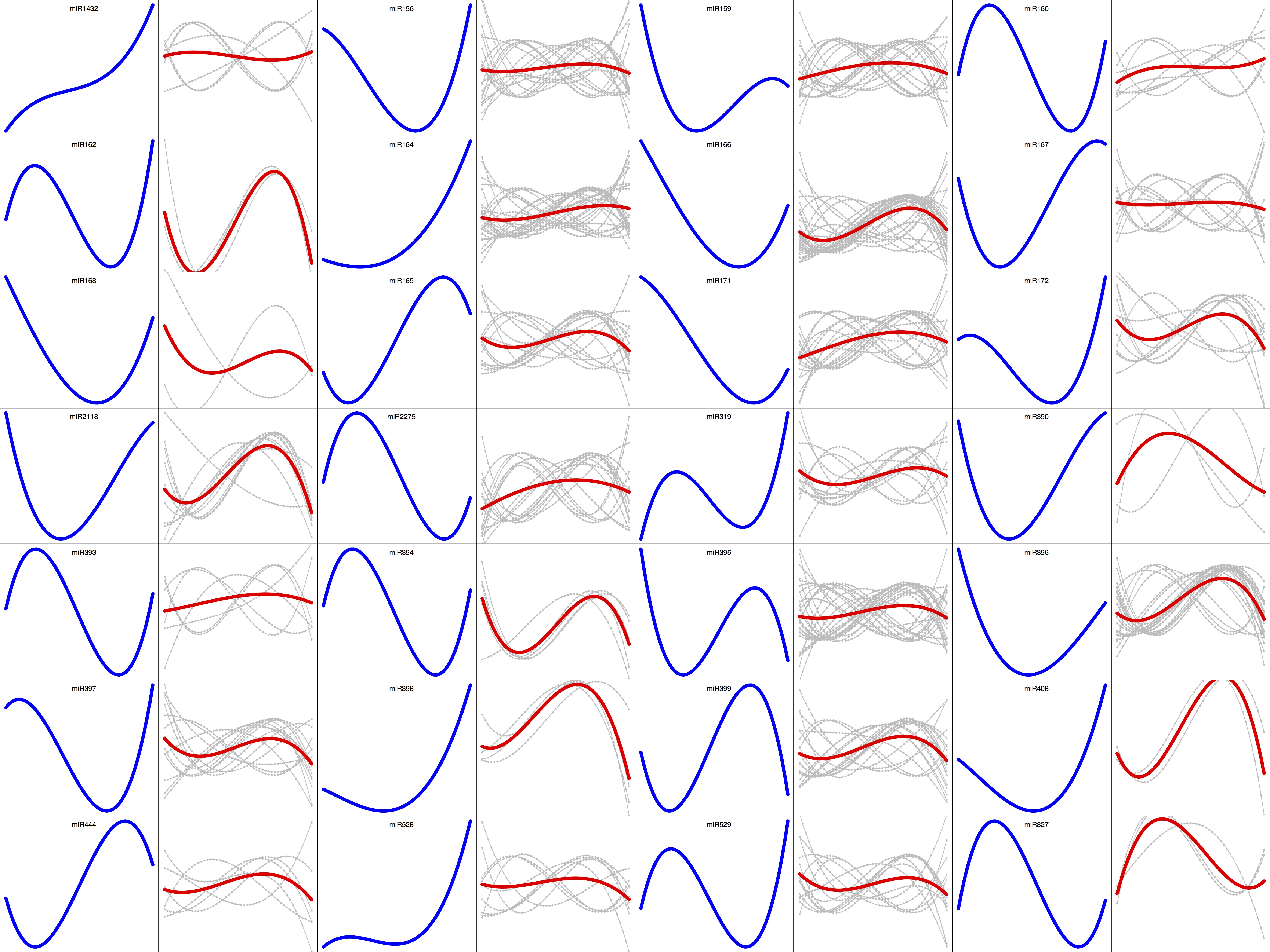

Supplement: Supplementary file 17 — Additional file 17. Expression patterns of conserved miRNAs and their predicted target genes. TPQ (transcripts per quarter million) values for all 7 time points were regressed using 3rd order polynomial regression. For Zea mays (A) and Oryza sativa (B), blue lines represent expression patterns of miRNAs. Grey lines represent expression patterns of their corresponding predicted target genes. Average values were plotted as red lines. [file 13104_2016_2367_MOESM17_ESM.zip › Additional file 4 page 1.jpg]

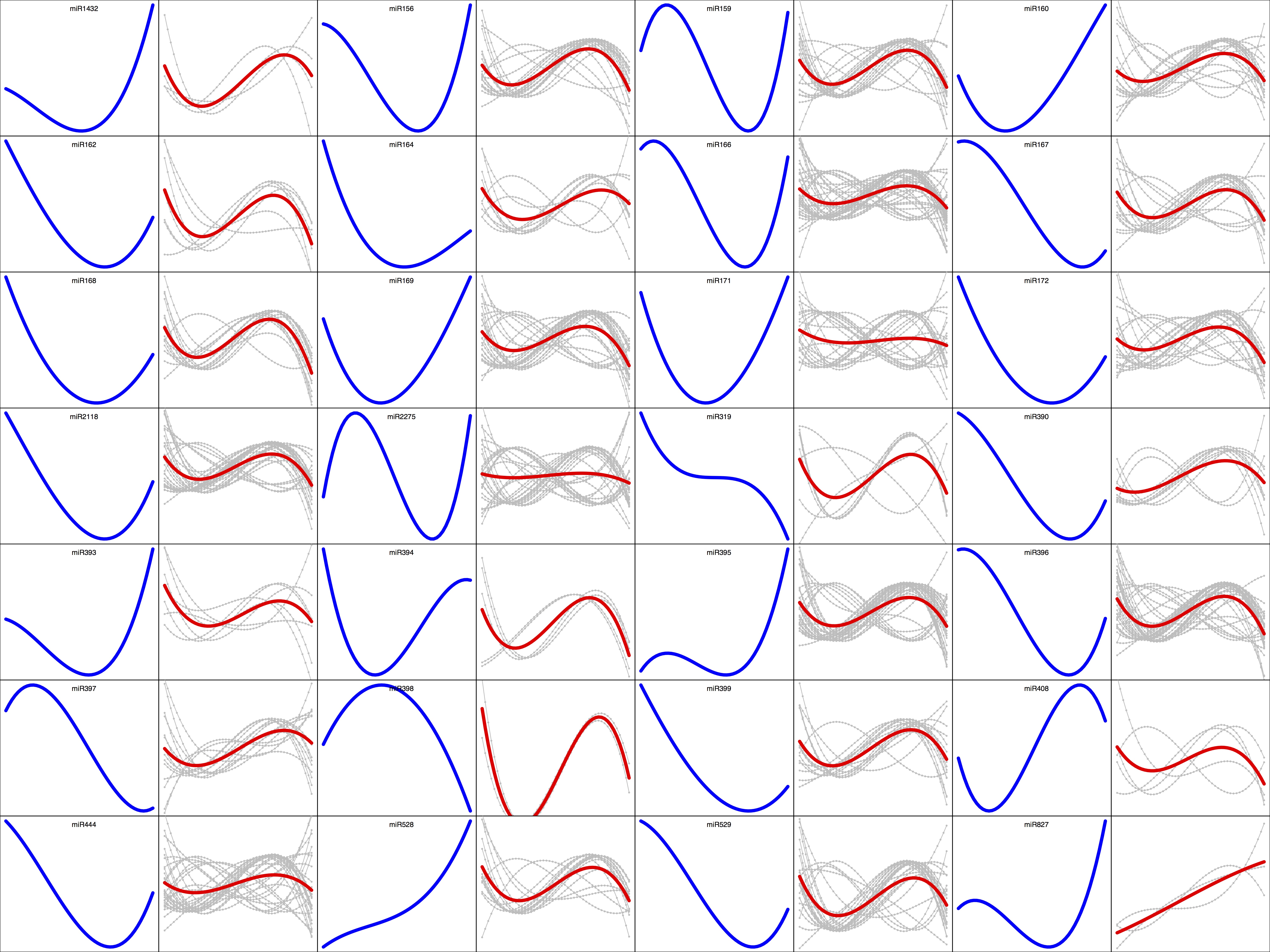

Supplement: Supplementary file 17 — Additional file 17. Expression patterns of conserved miRNAs and their predicted target genes. TPQ (transcripts per quarter million) values for all 7 time points were regressed using 3rd order polynomial regression. For Zea mays (A) and Oryza sativa (B), blue lines represent expression patterns of miRNAs. Grey lines represent expression patterns of their corresponding predicted target genes. Average values were plotted as red lines. [file 13104_2016_2367_MOESM17_ESM.zip › Additional file 4 page 2.jpg]
